# AI for evidence-based treatment recommendation in oncology: a blinded evaluation of large language models and agentic workflows

**DOI:** 10.3389/frai.2025.1683322

**Published:** 2025-12-09

**Authors:** Guannan Zhai, Merav Bar, Andrew J. Cowan, Samuel Rubinstein, Qian Shi, Ningjie Zhang, En Xie, Will Ma

**Affiliations:** 1 Department of Statistics, George Washington University, Washington, DC, United States; 2 Bristol Myers Squibb, Summit, NJ, United States; 3 Division of Hematology-Oncology, University of Washington, Seattle, WA, United States; 4 Division of Clinical Research, Fred Hutch Cancer Center, Seattle, WA, United States; 5 Division of Hematology, University of North Carolina, Chapel Hill, NC, United States; 6 Department of Quantitative Health Sciences, Mayo Clinic, Rochester, MN, United States; 7 Department of Bioinformatics and Biostatistics, Shanghai Jiao Tong University, Shanghai, China; 8 HopeAI, Inc, Princeton, NJ, United States

**Keywords:** agentic workflows, artificial intelligence in medicine, clinical evaluation, evidence-based medicine, large language models, multiple myeloma, oncology decision support, retrieval-augmented generation

## Abstract

**Background:**

Evidence-based medicine is crucial for clinical decision-making, yet studies suggest that a significant proportion of treatment decisions do not fully incorporate the latest evidence. Large Language Models (LLMs) show promise in bridging this gap, but their reliability for medical recommendations remains uncertain.

**Methods:**

We conducted an evaluation study comparing five LLMs’ recommendations across 50 clinical scenarios related to multiple myeloma diagnosis, staging, treatment, and management, using a unified evidence cutoff of June 2024. The evaluation included three general-purpose LLMs (OpenAI o1-preview, Claude 3.5 Sonnet, Gemini 1.5 Pro), one retrieval-augmented generation (RAG) system (Myelo), and one agentic workflow-based system (HopeAI). General-purpose LLMs generated responses based solely on their internal knowledge, while the RAG system enhanced these capabilities by incorporating external knowledge retrieval. The agentic workflow system extended the RAG approach by implementing multi-step reasoning and coordinating with multiple tools and external systems for complex task execution. Three independent hematologist-oncologists evaluated the LLM-generated responses using standardized scoring criteria developed specifically for this study. Performance assessment encompassed five dimensions: accuracy, relevance, comprehensiveness, hallucination rate, and clinical use readiness.

**Results:**

HopeAI demonstrated superior performance across accuracy (82.0%), relevance (85.3%), and comprehensiveness (74.0%), compared to OpenAI o1-preview (64.7, 57.3, 36.0%), Claude 3.5 Sonnet (50.0, 51.3, 29.3%), Gemini 1.5 Pro (48.0, 46.0, 30.0%), and Myelo (58.7, 56, 32.7%). Hallucination rates were consistently low across all systems: HopeAI (5.3%), OpenAI o1-preview (3.3%), Claude 3.5 Sonnet (10.0%), Gemini 1.5 Pro (8.0%), and Myelo (5.3%). Clinical use readiness scores were relatively low for all systems: HopeAI (25.3%), OpenAI o1-preview (6.0%), Claude 3.5 Sonnet (2.7%), Gemini 1.5 Pro (4.0%), and Myelo (4.0%).

**Conclusion:**

This study demonstrates that while current LLMs show promise in medical decision support, their recommendations require careful clinical supervision to ensure patient safety and optimal care. Further research is needed to improve their clinical use readiness before integration into oncology workflows. These findings provide valuable insights into the capabilities and limitations of LLMs in oncology, guiding future research and development efforts toward integrating AI into clinical workflows.

## Introduction

For treatment decision making, physicians typically rely on clinical practice guidelines, such as National Comprehensive Cancer Network (NCCN) (Kumar et al., 2025), American Society of Clinical Oncology (ASCO) guidelines in the United States (Dimopoulos et al., 2021), and European Society of Medical Oncology (ESMO) guidelines in Europe (Mikhael et al., 2019). However, these guidelines may still lag behind the most current evidence due to the rapid emergence of new therapies in oncology (Flora et al., 2023; Kraeber-Bodéré et al., 2023). For example, patients with relapsed or refractory multiple myeloma now have access to several approved therapeutic options - such as CAR-T cells, bispecific antibodies, and novel agent combination regimens (Munshi et al., 2021; Parrondo et al., 2024; Sheykhhasan et al., 2024) - which have led to significant improvements in outcomes (Turesson et al., 2010). The fast-paced evolution of treatments, frequent updates to guidelines, and variations across regions, have created a disconnect, where oncologists and hematologists may not always have the most relevant and current information to help their decisions. This disconnect makes it increasingly difficult for these clinicians to stay current with evidence-based practices (Bar et al., 2023; Cowan et al., 2022; Garfall, 2024; Rodriguez-Otero et al., 2024; Subbiah, 2023), and forces them to navigate an increasingly complex information landscape, a challenge further compounded by the projected global shortage of hematologist-oncologists (Yang et al., 2014).

Since 2022, large language models (LLMs) have made remarkable advances, demonstrating human-like reasoning capabilities (Achiam et al., 2023; Chowdhery et al., 2023; Bubeck et al., 2023). Although these models show promise (Biswas, 2023; Benary et al., 2023; Lee et al., 2023; Gilbert et al., 2023), recent studies suggest that about one-third of LLM-generated cancer treatment recommendations may contain potentially harmful inaccuracies, underscoring the need for caution when deploying these systems in clinical practice (Karabacak and Margetis, 2023). Moreover, LLMs face inherent context limitations that prevent them from processing entire sets of guidelines at once (Hadi et al., 2023).

To address these challenges, newer approaches such as Retrieval Augmented Generation (RAG) (Quidwai and Lagana, 2024) and agentic workflow (Li et al., 2024; Singh et al., 2024) have emerged, aiming to enhance the accuracy and reliability of LLM-derived medical recommendations. These innovations seek to mitigate limitations like hallucinations and context constraints, thereby improving the integration of LLMs into evidence-based clinical decision-making. In the present study, we evaluated five LLM models - three general-purpose LLMs (OpenAI o1-preview, Claude 3.5 Sonnet, Gemini 1.5 Pro), one RAG-based model (Myelo), and one agentic workflow (HopeAI) - to determine their effectiveness in delivering accurate, evidence-based treatment recommendations for multiple myeloma. By examining the relative strengths and limitations of these methods, our research aims to contribute to the ongoing effort to develop dependable and relevant AI-assisted clinical decision support tools that help bridge the gap between emerging medical evidence and real-world patient care.

## Methods

### Clinical scenarios

This study used a set of 50 clinical scenarios covering various aspects of multiple myeloma. These scenarios were developed by a panel of three hematologist-oncologists specializing in clinical diagnosis and treatment, and were reviewed to ensure clarity and clinical relevance. The 50 clinical scenarios were categorized into five domains: (1) multiple myeloma diagnostic assessment, (2) treatment recommendations for newly diagnosed multiple myeloma (NDMM), (3) treatment recommendations for relapsed/refractory multiple myeloma (RRMM), (4) management of special clinical scenarios, and (5) novel therapeutics. (A full list of 50 clinical scenarios is provided in the Supplementary material).

### Large language models

In this study, we evaluated five AI systems for evidence-based treatment recommendation: OpenAI o1-preview, Claude 3.5 Sonnet, Gemini 1.5 Pro, Myelo, and HopeAI. Our goal was to compare three distinct system-level approaches: General-Purpose Large Language Models (LLMs), Retrieval-Augmented Generation (RAG), and Agentic Workflows (Agents). Crucially, this was a comparison of systems in their typical deployment, not a direct benchmark of the underlying models.

#### General-purpose large language models (LLMs)

These models process a given question or prompt directly using the knowledge encoded during their pretraining. They generate responses only based on the internal knowledge without accessing any external data sources. Three general-purpose LLMs were included in this study:

*OpenAI o1-preview*, developed by OpenAI, released on September 12, 2024 (Introducing OpenAI o1-Preview, 2024).

*Claude 3.5 Sonnet*, developed by Anthropic, released in June 2024 (Anthropic, 2024).

*Gemini 1.5 Pro*, developed by Google DeepMind and released in February 2024 (Google DeepMind, 2024).

#### Retrieval-augmented generation (RAG)

RAG expands on what general-purpose LLMs can accomplish by incorporating a knowledge retrieval step that pulls from external sources. One RAG-based LLM was included in this study:

*Myelo*, a disease-specific chatbot developed by the International Myeloma Foundation^1^ in collaboration with ZS Associates and Amazon Web Services (AWS). It is designed to provide compassionate support for multiple myeloma patients, caregivers, and health professionals.

#### Agentic workflows (agent)

Agentic workflows take the RAG approach a step further by incorporating multi-step reasoning and task execution. Beyond retrieving external data, agents coordinate with multiple tools, APIs, or external systems to complete complex tasks. One Agent was included in this study:

*HopeAI* employs an agentic workflow to extract, curate, and standardize clinical evidence from both primary and secondary clinical trial publications. The evidence is synthesized using statistical analysis methods, including meta-analysis and pooled analysis.

### Inference settings

Unless otherwise specified, all generations used temperature = 0.7 and top *p* = 1.0. These values were selected to balance stability (comparability across systems) and output diversity. The same settings were applied to general-purpose LLMs, the RAG system (Myelo), and the agentic workflow (HopeAI) at the response-generation step.

### Evidence currency controls

All model outputs were generated in June 2024, which served as the unified evidence cutoff for the evaluation. Each system was used in its production configuration available at that time.

Claude 3.5 Sonnet has publicly stated training data extending into early 2024 and supports real-time web search. OpenAI has not published a formal knowledge-cutoff date for the o1-preview model; it was evaluated as deployed in June 2024, including its real-time search capability. Google has likewise not released a specific knowledge-cutoff date for Gemini 1.5 Pro, although public technical documentation indicates training data extending into early 2024, and the model integrates search features. For HopeAI and Myelo, no training cutoff information is publicly available, and both systems were evaluated in their June 2024 production versions.

Three hematologist–oncologists reviewed all model responses using guideline evidence expected to be current as of June 2024. Because the study was conducted prospectively at a single timepoint, all systems had contemporaneous access to the evidence available by the cutoff month.

### Blinded evaluation

The five LLMs’ responses to the clinical scenarios were evaluated by three hematologist-oncologists, each with extensive experience in multiple myeloma. During the assessment process, evaluators were blinded to which LLM generated each response. Specifically, model names, metadata, and formatting features were removed, and all texts were standardized to a uniform font and layout. Evaluators were informed only that responses originated from different AI systems. No evaluator reported recognizing or suspecting model identity during the assessment. To ensure comparability across systems, all evaluations were performed using evidence current through June 2024, reflecting the same knowledge and regulatory context for every model.

The evaluation framework encompassed five predefined dimensions for performance evaluations: accuracy, relevance/timeliness, comprehensiveness, hallucination and ready-to-use status. The first three were assessed using 4-point scales, ranging from complete fulfillment (4) to incomplete fulfillment (1). For example, for accuracy: 4-completely accurate, 3-mostly accurate, 2-partially accurate, and 1-inaccurate. The last two were evaluated as binary outcomes, indicating the presence or absence of fabricated information and the need or no need for editing before clinical use, respectively.

Among the five evaluation dimensions, *accuracy* was considered the primary endpoint, as it most directly reflects the alignment of model-generated recommendations with evidence-based clinical guidelines. The remaining dimensions were treated as independent descriptive metrics to provide complementary perspectives on model performance.

### Analysis methods

#### Inter-evaluator agreement analysis

Agreement among three evaluators was assessed through three approaches: weighted Cohen’s Kappa analysis (Blackman and Koval, 2000), intra-class correlation (ICC) analysis, and disagreement scenario analysis.

For the 4-point rating scales (accuracy, relevance, comprehensiveness), weighted Cohen’s Kappa was calculated to quantify pairwise agreement between evaluators. This metric accounts for chance agreement and yields coefficients from −1 to +1, where −1 indicates complete disagreement, 0 indicates chance-level agreement, and +1 indicates perfect agreement. Linear weights were applied (1.0 for perfect agreement, 0.667 for one-point differences, 0.333 for two-point differences, and 0 for three-point differences). Pairwise Kappa values were calculated separately for each dimension per each model.

In addition, intra-class correlation coefficients [ICC (Dimopoulos et al., 2021; Kumar et al., 2025) and ICC (Dimopoulos et al., 2021; Mikhael et al., 2019)] were computed using a two-way random-effects, absolute-agreement model to evaluate the reliability of ratings across all three evaluators. ICC(2,1) represents single-rater reliability, capturing scenario-level variance components, while ICC(2,3) quantifies the reliability of the averaged ratings. Variance components for scenario, rater, and residual effects were estimated using restricted maximum likelihood and summarized as proportions of total variance.

To supplement the Kappa analysis and ICC analyses, disagreement scenarios were analyzed. A disagreement scenario was operationally defined as any instance where the maximum score difference among the three evaluators exceeds one point on the 4-point scale. The frequency of disagreement scenarios was calculated as a percentage of total evaluations for each dimension per each model.

For binary dimensions (hallucination and ready-to-use status), Cohen’s Kappa was not utilized owing to its known limitations with binary ratings, including paradoxical behavior in settings with high observed agreement (Cicchetti and Feinstein, 1990). Instead, disagreement scenario analysis was conducted to assess agreement.

#### Quantitative analysis

The performance of each model was evaluated across five dimensions (accuracy, relevance/timeliness, comprehensiveness, hallucination rate, and ready-to-use without editing) based on 50 clinical scenarios, each rated by three evaluators. For each response generated by a given LLM in a specific clinical scenario, evaluators assessed the response and scored it according to predefined rating levels. (e.g., a 4-point rating scale was used to assess accuracy, relevance/timeliness, and comprehensiveness, while binary (0/1) scoring was applied for hallucination and ready-to-use status.).

For each LLM and each rating dimension, the rating distribution is computed by first determining, for each evaluator, the proportion of responses that received each score across 50 clinical scenarios, and then averaging these proportions across three evaluators to obtain the final aggregated percentages. This methodology was applied uniformly across all dimensions and models.

To quantify uncertainty for each rating dimension, we further used scenario-level nonparametric bootstrap resampling. Scores were first averaged across the three blinded evaluators within each clinical scenario. We then generated 5,000 bootstrap samples at the scenario level, treating each scenario as a single sampling unit so that all system scores from the same case were resampled together. For each bootstrap sample, the mean score of each system was recalculated, and percentile-based 95% confidence intervals (Cls) were derived from the 2.5^th^ and 97.5^th^ percentiles of the bootstrap distribution. To examine overall differences between systems, we applied the Friedman test to the ordinal metrics (accuracy, relevance, and comprehensiveness) using paired observations across all scenario-reviewer combinations, with statistical significance set at p<0.05 .

In addition to the five-evaluation dimension, for each of the 50 clinical scenarios, evaluators also ranked the responses provided by five LLMs (blinded to the model names: OpenAI o1-preview, Claude 3.5 sonnet, Gemini 1.5 Pro, HopeAI, and Myelo) from first to fifth based on overall quality. The ranking distribution for each model was calculated by dividing the frequency of each rank position (across all scenarios and evaluators) by the total number of evaluations (150 rankings per model; 50 scenarios × 3 evaluators). This analysis enabled assessment of relative model performance, as demonstrated in the accompanying visualizations.

#### Qualitative analysis

A systematic review of evaluator comments was conducted to identify recurring patterns in evaluating the model performance. The analysis examined model limitations, model-specific strengths and weaknesses, and systematic gaps in treatment recommendations. Emphasis was placed on regulatory compliance and currency of clinical knowledge, specifically examining instances of regulatory misalignment and recommendations for inappropriate or withdrawn treatments. Cross-model performance analysis synthesized comparative data across all evaluated dimensions to characterize model-specific patterns in clinical scenario management.

## Results

The blinded evaluation of five LLMs in the multiple myeloma domain generated 4,500 assessment data points. This dataset was derived from presenting 50 clinical scenarios to five LLMs (Gemini 1.5 Pro, Claude 3.5 Sonnet, OpenAI o1-preview, Myelo, and HopeAI), with each response produced by each LLM independently evaluated by three blinded evaluators across six evaluation dimensions: accuracy, relevance/timeliness, comprehensiveness, hallucination rate, ready-to-use without editing, and overall rank.

### Inter-evaluator agreement

Evaluators’ inter-evaluator agreement varied substantially across models and evaluation dimensions. For accuracy assessment, Myelo demonstrated the strongest agreement (*κ* = 0.71) among three evaluators, while other models showed moderate to minimal agreement (*κ* ranging from 0.58 to 0.04). Similar patterns were observed in relevance/timeliness assessment, with Myelo achieving the highest agreement (κ = 0.81), followed by Claude 3.5 Sonnet (κ = 0.61). Comprehensiveness evaluations generally showed lower agreement for all models (κ ranging from 0.64 to 0.11) among three evaluators.

To complement these results, we computed ICC(2,3) and ICC(2,1) to assess inter-rater reliability under a two-way random-effects, absolute-agreement model. Across systems and evaluation dimensions, ICC(2,3) ranged from 0.03 to 0.61 and ICC(2,1) from 0.01 to 0.34, consistent with the moderate reliability typically observed in subjective clinical assessments. Variance decomposition based on the same model indicated that scenario-level variance accounted for 0.1–35% of total variance, rater-level variance for 15–45%, and residual variance for 40–60%. Systems such as Myelo and Claude 3.5 Sonnet showed higher scenario-level variance and lower rater-level variance, corresponding to greater consistency across evaluators.

In contrast, HopeAI exhibited a distinct reliability pattern. Although it received among the highest average scores, its ICC values were lower due to compressed rating ranges and greater between-rater variance. Specifically, only 0–6% of total variance was attributed to scenario differences, while 35–45% originated from rater-level variation. This suggests that one evaluator applied a more conservative scoring scale, while others were slightly more generous. Because most HopeAI responses were rated near the top of the scale (e.g., 3–4 on a 4-point scale), small differences in scoring thresholds were amplified mathematically, resulting in lower ICC values even when evaluators broadly agreed on overall quality.

Disagreement scenario analysis aligned with these findings. The proportion of scenarios with substantial evaluator differences was consistently lower for Myelo (6.0 to 19.6% across dimensions) and higher for HopeAI (24.0 to 45.1%).

The rank correlation analysis demonstrated moderate consistency in overall ranking of responses produced by 5 LLMs within each evaluator (correlation coefficient = 0.498, *p* < 0.0001). This significant correlation suggests that despite variations in individual ratings and some disagreements across different dimensions, evaluators showed reasonable agreement in their relative ranking of these five LLM models’ overall performance across 50 clinical scenarios.

The patterns of evaluator variability across all five dimensions are presented in Table 1.

**Table 1:** Inter-evaluator agreement among three evaluators.

Dimension	Model	Average Kappa	ICC(2,3)^+^	ICC(2,1)*	Disagreement
Accuracy	Myelo	0.71	0.59	0.33	4 scenarios (8.0%)
	OpenAI o1-preview	0.55	0.36	0.16	10 scenarios (19.6%)
	Gemini 1.5 Pro	0.26	0.4	0.18	6 scenarios (12.0%)
	Claude 3.5 Sonnet	0.58	0.56	0.30	7 scenarios (14.0%)
	HopeAI	0.04	0.03	0.01	16 scenarios (32.0%)
Relevance	Myelo	0.81	0.61	0.34	3 scenarios (6.0%)
	OpenAI o1-preview	0.54	0.47	0.23	9 scenarios (17.6%)
	Gemini 1.5 Pro	0.19	0.34	0.15	14 scenarios (28.0%)
	Claude 3.5 Sonnet	0.61	0.48	0.23	4 scenarios (8.0%)
	HopeAI	0.17	0.15	0.06	12 scenarios (24.0%)
Comprehensive	Myelo	0.64	0.52	0.27	10 scenarios (19.6%)
	OpenAI o1-preview	0.39	0.47	0.23	16 scenarios (31.4%)
	Gemini 1.5 Pro	0.40	0.51	0.26	15 scenarios (29.4%)
	Claude 3.5 Sonnet	0.56	0.53	0.27	10 scenarios (19.6%)
	HopeAI	0.11	0.11	0.10	23 scenarios (45.1%)

^+^ICC(2,3): Two-way random-effects, absolute-agreement model for the average of three raters. ^*^ICC(2,1): Two-way random-effects, absolute-agreement model for single-rater reliability.

For the two binary evaluation dimensions (hallucination and ready-to-use), inter-rater consistency was assessed using both scenario-level disagreement rates and formal reliability statistics. Across the five evaluated systems, disagreement on hallucination ranged from 8.0 to 30.0%, corresponding to overall agreement levels of approximately 70–92%. Specifically, disagreement occurred in 8.0% of cases for OpenAI o1-preview, 14.0% for HopeAI, 14.0% for Myelo, 24.0% for Gemini 1.5 Pro, and 30.0% for Claude 3.5 Sonnet. To more formally quantify agreement while accounting for the highly imbalanced distribution of hallucination ratings (the vast majority labeled “no hallucination”), we used Gwet’s AC1, which is more stable than Fleiss’ *κ* under prevalence asymmetry. Hallucination exhibited high agreement (AC1 = 0.86, 95% CI: [0.82–0.91]).

**Figure 1:**
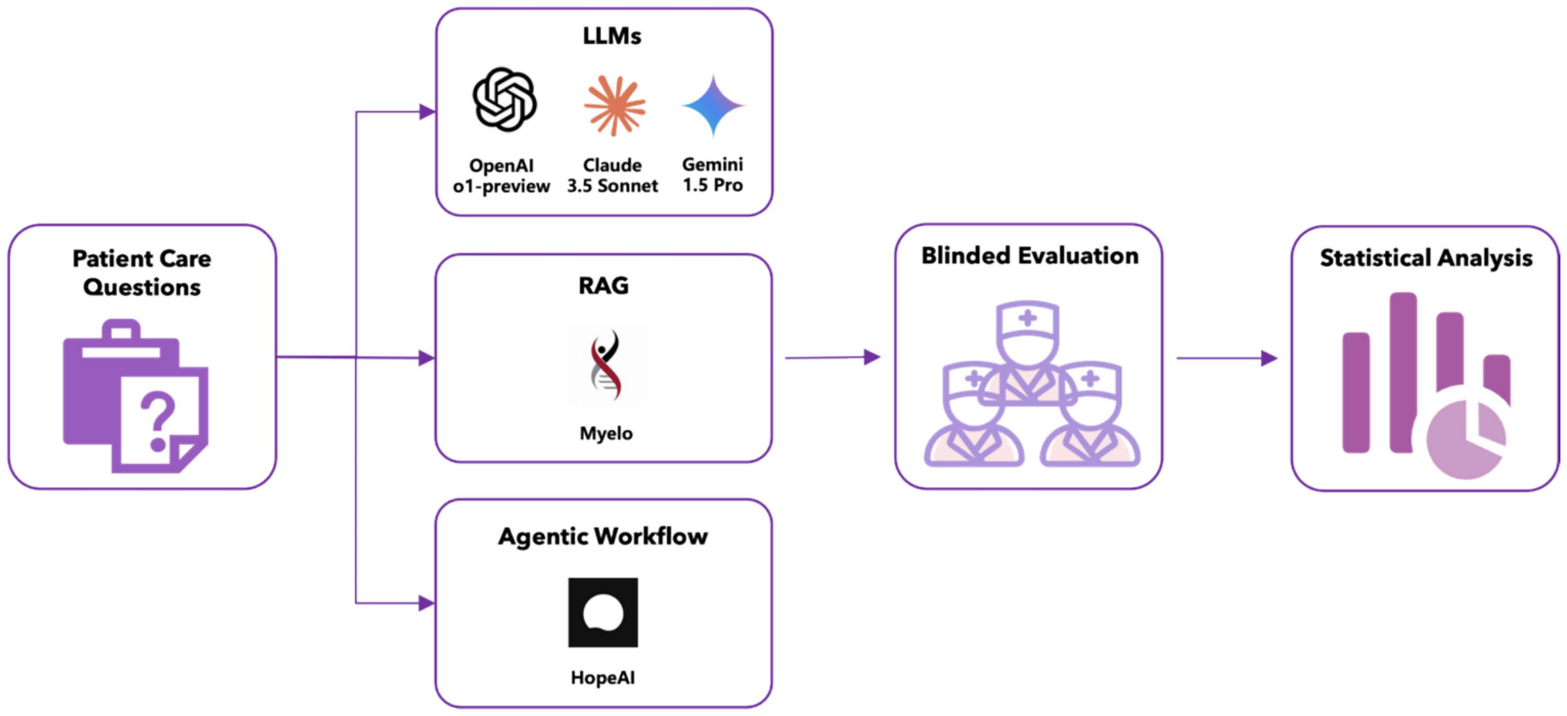
Workflow for comparative study of LLMs.

For the ready-to-use dimension, disagreement rates were substantially higher, ranging from 12.0 to 64.0% across models. Myelo showed the lowest disagreement (12.0%), followed by Gemini 1.5 Pro (14.0%), Claude 3.5 Sonnet (16.0%), and OpenAI o1-preview (26.0%), while HopeAI demonstrated the highest rate (64.0%). Correspondingly, Gwet’s AC1 indicated moderate-to-substantial agreement for this binary outcome (AC1 = 0.77, 95% CI: [0.71–0.83]). Taken together, the disagreement frequencies and AC1 statistics provide a consistent picture: hallucination judgments were highly concordant across raters, whereas ready-to-use ratings, which inherently require stricter clinical standards, exhibited more variability (Figure 1).

### Quantitative analysis results

#### Accuracy

**Figure 2:**
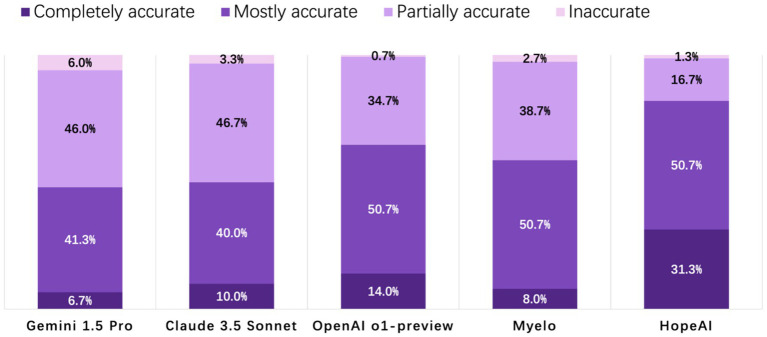
Evaluation of LLM-generated recommendations across the accuracy dimension (4-point scale: 4 = completely accurate, 3 = mostly accurate, 2 = partially accurate, 1 = inaccurate).

HopeAI responses were rated as completely accurate for 31.3% of the 50 clinical scenarios, while OpenAI o1-preview achieved 14.0%, followed by Claude 3.5 Sonnet (10.0%), Myelo (8.0%), and Gemini 1.5 Pro (6.7%). When considering both completely and mostly accurate responses combined, HopeAI responses were rated as mostly to completely accurate for 82.0% of the 50 clinical scenarios, followed by OpenAI o1-preview (64.7%), Myelo (58.7%), Claude 3.5 Sonnet (50.0%), and Gemini 1.5 Pro (48.0%) (Figure 2).

#### Relevance / timeliness

**Figure 3:**
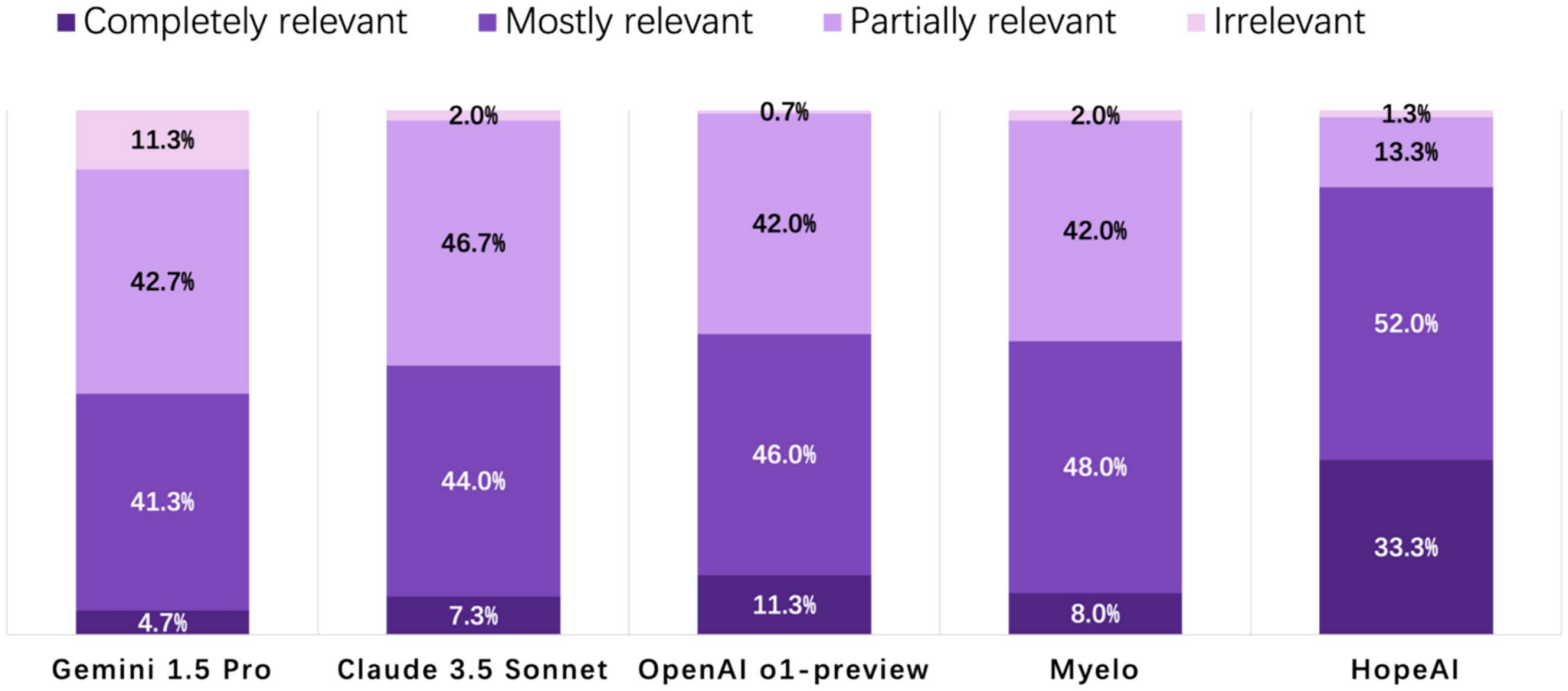
Evaluation of LLM-generated recommendations across the relevance/timeliness dimension (4-point scale: 4 = highly relevant and up-to-date, 3 = mostly relevant, 2 = partially relevant or outdated, 1 = irrelevant).

HopeAI responses were rated as completely relevant for 33.3% of the 50 clinical scenarios, while OpenAI o1-preview achieved 11.3%, followed by Myelo (8.0%), Claude 3.5 Sonnet (7.3%), and Gemini 1.5 Pro (4.0%). When considering both completely and mostly relevant responses combined, HopeAI responses were rated as mostly to completely relevant for 85.3% of the 50 clinical scenarios (33.3% completely + 52.0% mostly relevant), followed by OpenAI o1-preview (57.3%), Myelo (56.0%), Claude 3.5 Sonnet (51.3%), and Gemini 1.5 Pro (46.0%) (Figure 3).

#### Comprehensiveness

**Figure 4:**
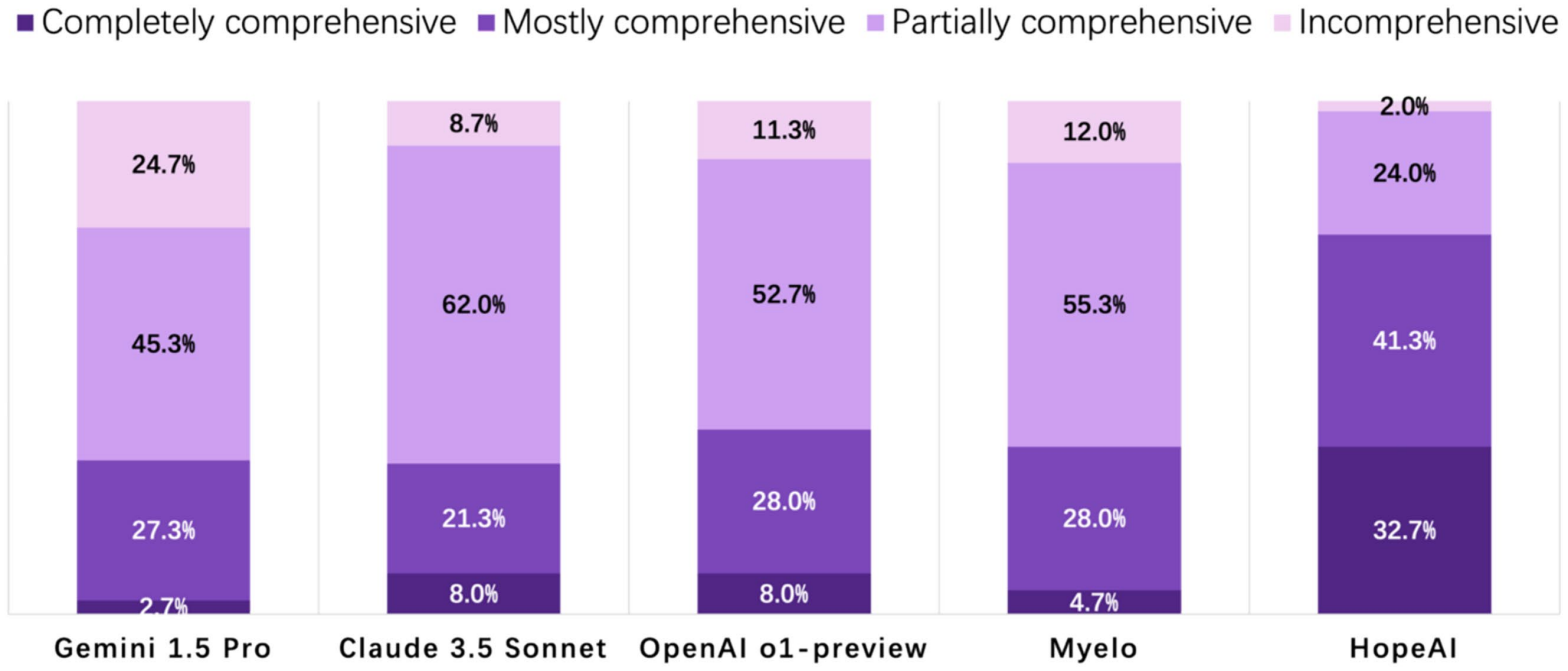
Evaluation of LLM-generated recommendations across the comprehensiveness dimension (4-point scale: 4 = fully comprehensive, 3 = mostly comprehensive, 2 = partially comprehensive, 1 = incomplete).

HopeAI responses were rated as completely comprehensive for 32.7% of the 50 clinical scenarios, while both OpenAI o1-preview and Claude 3.5 Sonnet achieved 8.0%, followed by Myelo (4.7%), and Gemini 1.5 Pro (2.7%). When considering both completely and mostly comprehensive responses combined, HopeAI responses were rated as mostly to completely comprehensive for 74.0% of the 50 clinical scenarios (32.7% completely + 41.3% mostly comprehensive), followed by OpenAI o1-preview (36.0%), Gemini 1.5 Pro (30.0%), and Myelo (29.3%) (Figure 4).

#### Hallucination

**Figure 5:**
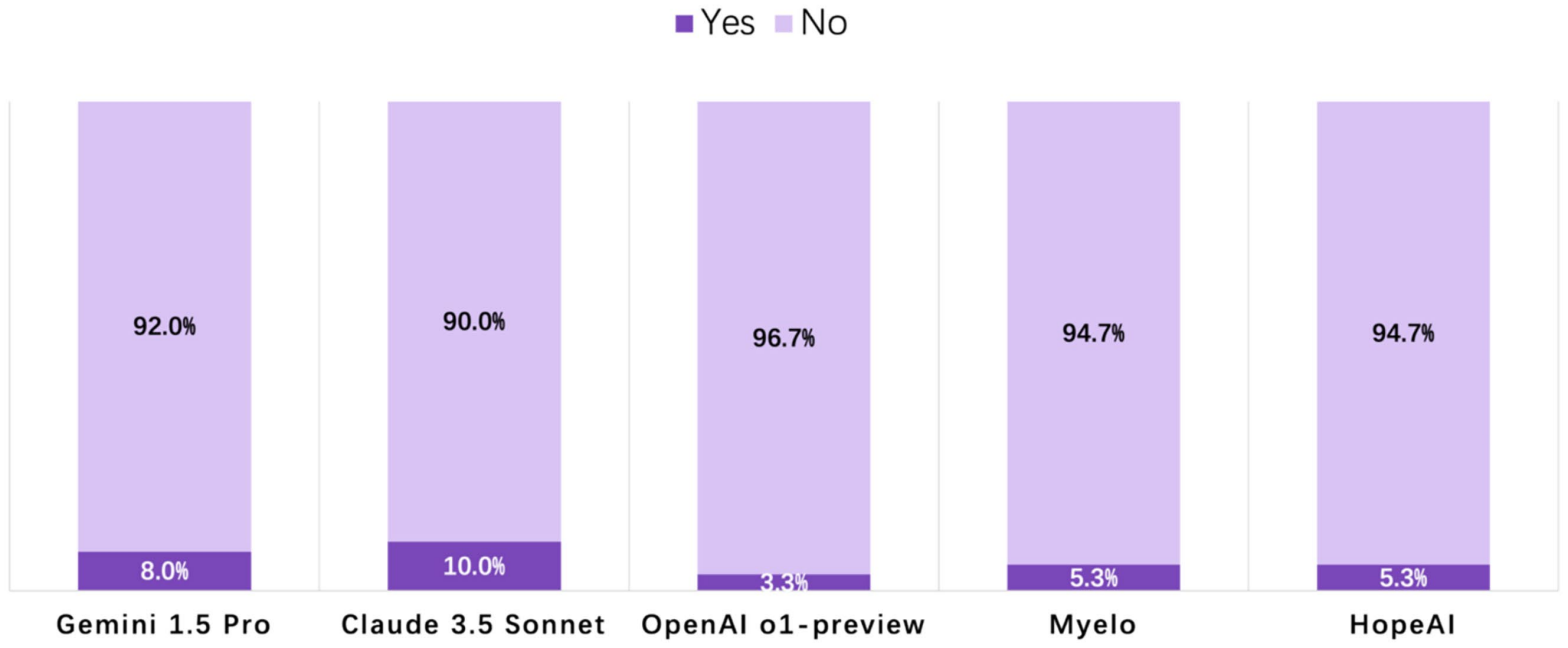
Evaluation of LLM-generated recommendations across the hallucination dimension (binary outcome: 1 = presence of hallucination, 0 = no hallucination).

Across the 50 clinical scenarios, hallucination rates ranged from 3.3 to 10.0% among the five LLMs, indicating that only a small proportion of responses contained fabricated or unsupported information. OpenAI o1-preview responses were rated at the lowest hallucination rate of 3.3%, while HopeAI and Myelo both showed a 5.3% hallucination rate. Claude 3.5 Sonnet and Gemini 1.5 Pro showed slightly higher rates at 10.0 and 8.0%, respectively, (Figure 5).

#### Clinical use readiness

**Figure 6:**
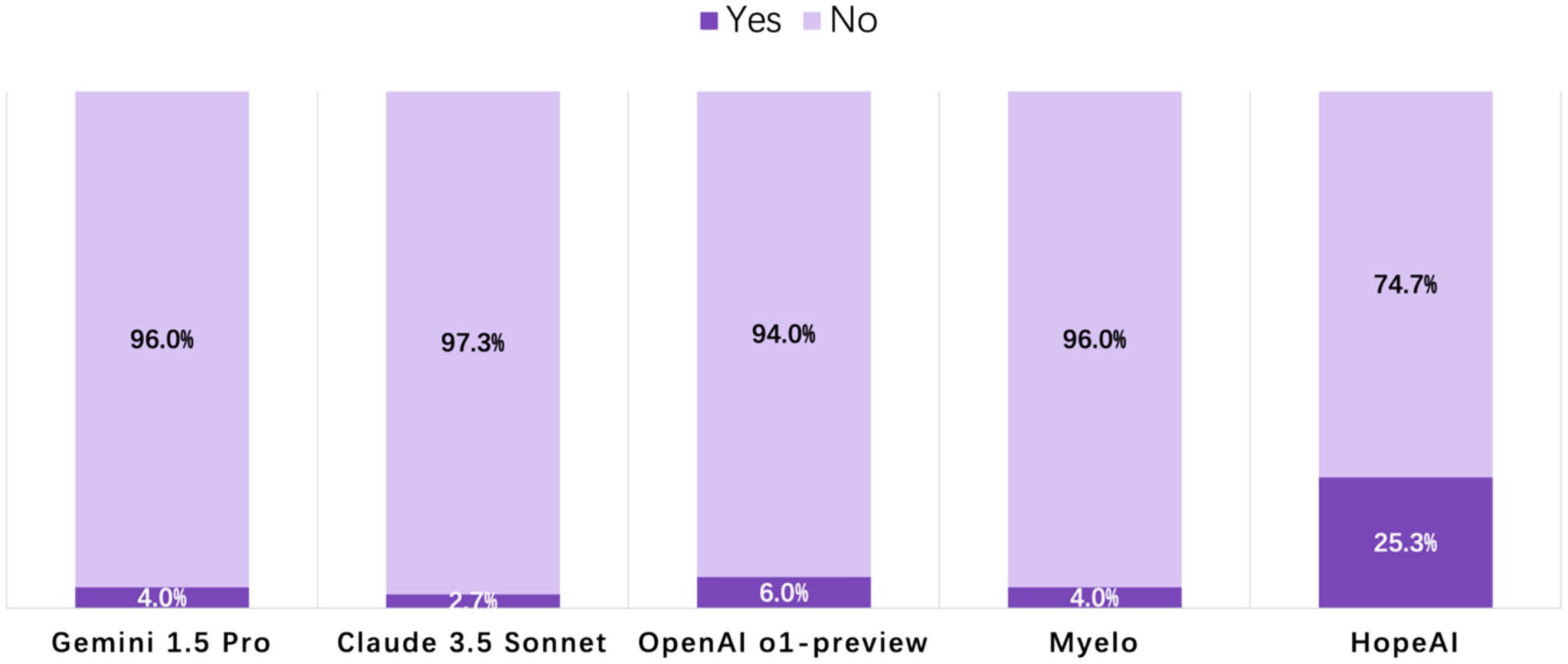
Evaluation of LLM-generated recommendations across the clinical-use readiness dimension (binary outcome: 1 = ready for immediate clinical use, 0 = requires editing or verification).

Analysis of clinical readiness revealed that most LLMs’ responses required editing before implementation. The highest proportion of HopeAI responses were rated as immediately usable responses (25.3%), followed by OpenAI o1-preview (6.0%), Myelo (4.0%), Gemini 1.5 Pro (4.0%), and Claude 3.5 Sonnet (2.7%) (Figure 6).

#### Statistical comparison and uncertainty analysis

Following the approach described above, scenario-level bootstrap analyses were conducted to estimate 95% confidence intervals (CIs) for each evaluation metric. The resulting mean scores and confidence intervals for accuracy, relevance, comprehensiveness, hallucination, and ready-to-use are summarized in Table 2.

**Table 2:** Performance of Five AI systems across evaluation dimensions.

Metric	Scale definition	System	Mean (95% CI)
Accuracy	4 = completely accurate; 3 = mostly accurate; 2 = partially accurate; 1 = inaccurate	HopeAI	3.12 (3.02–3.22)
		OpenAI o1-preview	2.78 (2.67–2.90)
		Myelo	2.64 (2.51–2.77)
		Claude 3.5 Sonnet	2.57 (2.43–2.70)
		Gemini 1.5 Pro	2.49 (2.37–2.61)
Relevance	4 = completely accurate; 3 = mostly accurate; 2 = partially accurate; 1 = inaccurate	HopeAI	3.17 (3.07–3.27)
		OpenAI o1-preview	2.68 (2.55–2.81)
		Myelo	2.62 (2.49–2.75)
		Claude 3.5 Sonnet	2.57 (2.45–2.69)
		Gemini 1.5 Pro	2.39 (2.27–2.52)
Comprehensiveness	4 = fully comprehensive; 3 = mostly comprehensive; 2 = partially comprehensive; 1 = incomplete	HopeAI	3.05 (2.95–3.15)
		OpenAI o1-preview	2.33 (2.18–2.47)
		Myelo	2.25 (2.12–2.39)
		Claude 3.5 Sonnet	2.29 (2.15–2.43)
		Gemini 1.5 Pro	2.08 (1.93–2.23)
Hallucination	Binary 1 = presence of hallucination; 0 = no hallucination	HopeAI	0.05 (0.02–0.09)
		OpenAI o1-preview	0.03 (0.01–0.07)
		Myelo	0.05 (0.02–0.09)
		Claude 3.5 Sonnet	0.10 (0.06–0.14)
		Gemini 1.5 Pro	0.08 (0.04–0.12)
Clinical Use Readiness	Binary 1 = ready for immediate use; 0 = requires editing/verification	HopeAI	0.33 (0.25–0.41)
		OpenAI o1-preview	0.12 (0.06–0.19)
		Myelo	0.07 (0.02–0.13)
		Claude 3.5 Sonnet	0.07 (0.03–0.11)
		Gemini 1.5 Pro	0.07 (0.02–0.12)

As shown in Table 2, HopeAI achieved the highest mean scores across accuracy, relevance, comprehensiveness and clinical use readiness, with relatively narrow 95% confidence intervals indicating stable estimates. Similar patterns were observed for relevance and comprehensiveness. To formally test for overall differences among systems, Friedman tests were applied to the three ordinal metrics, yielding significant results for all (accuracy: *χ*^2^(4) = 112.15, *p* < 0.0001; relevance: *χ*^2^(4) = 123.85, *p* < 0.0001; comprehensiveness: *χ*^2^(4) = 169.14, *p* < 0.0001). These findings confirm that the observed performance differences among the five systems are statistically significant.

#### Overall ranking

**Figure 7:**
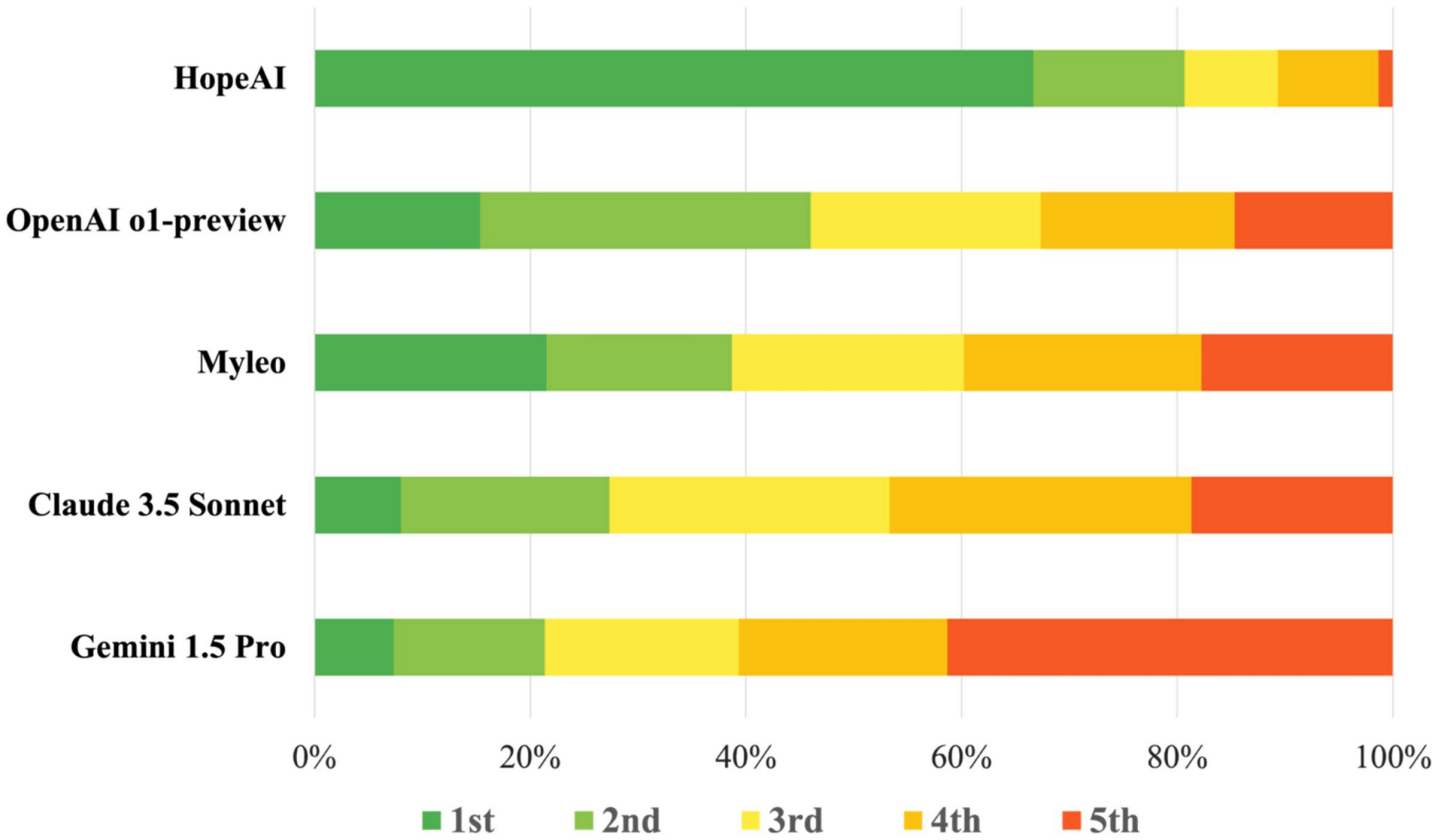
Ranking distribution of LLM-generated recommendations quality across models on 50 clinical scenarios.

In the comparative evaluation of LLM responses across 50 clinical scenarios by three independent reviewers (150 total evaluations), HopeAI responses were most frequently rated at top rankings, being rated first in 66.7% (100/150) of evaluations. Myelo received the second-highest proportion of first-place rankings (26.7%), followed by OpenAI o1-preview (15.3%), Claude 3.5 Sonnet (8.0%), and Gemini 1.5 Pro (7.3%) (Figure 7).

To formally assess ranking differences, we applied a non-parametric Friedman test based on scenario-level ranking scores. The test showed a significant overall difference among models (*χ*^2^ = 87.1, *p* < 0.0001), indicating that performance varied significantly across systems. Post-hoc pairwise Wilcoxon signed-rank tests with Holm correction confirmed significant ranking differences among several model pairs (*p* < 0.05). The tests indicated that HopeAI ranked significantly higher than the other systems in multiple comparisons, while OpenAI o1-preview also showed significant differences from several models (Table 3).

**Table 3:** *Post-hoc* pairwise comparisons of model rankings based on Wilcoxon signed-rank tests with Holm correction.

Model 1	Model 2	*p*-value (adjusted)	If significant
Claude 3.5 Sonnet	HopeAI	< 0.001	Yes
HopeAI	Myelo	< 0.001	Yes
Gemini 1.5 Pro	HopeAI	< 0.001	Yes
OpenAI o1-preview	HopeAI	< 0.001	Yes
OpenAI o1-preview	Gemini 1.5 Pro	0.0002	Yes
OpenAI o1-preview	Myelo	0.0063	Yes
OpenAI o1-preview	Claude 3.5 Sonnet	0.0475	Yes
Claude 3.5 Sonnet	Gemini 1.5 Pro	0.081	No
Gemini 1.5 Pro	Myelo	0.372	No
Claude 3.5 Sonnet	Myelo	0.414	No

### Qualitative analysis results

The clinical experts’ evaluation of the LLMs’ responses in multiple myeloma treatment scenarios identified four primary patterns of limitation: (1) incomplete integration of newly approved therapies, (2) misalignment with FDA-approved treatment lines, (3) recommendation of outdated or withdrawn treatments, and (4) inadequate consideration of treatment sequencing.

Firstly, analysis of recommendations for treatment for relapse after treatment with BCMA-targeted regimen demonstrated limited incorporation of newly approved therapies. In clinical scenarios where Talquetamab (GPRC5D-targeted therapy) represented an appropriate treatment option (Questions 13, 26, and 29), the therapy was frequently omitted from treatment considerations across all evaluated LLMs.

Second, analysis revealed misalignment with FDA-approved treatment lines, particularly for novel therapies, as evidenced in two primary categories:

Teclistamab and Elranatamab were incorrectly recommended for early-line treatment (in patients with 2–3 prior lines of treatment) while these agents are FDA-approved only for patients with at least 4 prior lines of therapy (5 L+). This pattern was observed in responses from OpenAI o1-preview (Q2), Claude 3.5 Sonnet (Q2), and HopeAI (Q2).

Ide-cel (Abecma) was inappropriately suggested for use in patients with one prior line of therapy (2 L), despite its FDA approval only for patients with at least 2 prior lines of therapy (3 L+). This misalignment was noted in responses from Myelo (Q15), OpenAI o1-preview (Q40), and Claude 3.5 Sonnet (Q39).

Third, analysis identified instances of recommendations for withdrawn or unapproved treatments. For example, belantamab mafodotin was recommended despite its market withdrawal, as observed in responses from Claude 3.5 Sonnet (Q6), OpenAI o1-preview (Q7), Myelo (Q19), and Gemini (Q35). Similarly, melflufen was suggested by OpenAI o1-preview and Claude 3.5 Sonnet despite its US market withdrawal. The DRVd regimen was recommended in responses from Myelo (Q21) and Claude 3.5 Sonnet (Q21), although FDA approval was still pending.

Fourth, evaluation revealed patterns of inadequate treatment sequencing consideration across three domains: (1) recognition of sequential same-target therapy implications, observed in all models except HopeAI in scenario Q30; (2) consideration of alternative targeting strategies, noted across all models in scenario Q32; and (3) discussion of bridging therapy for CAR-T candidates, particularly in responses from OpenAI o1-preview and Gemini in scenario Q42.

The detailed characteristics of each LLM’s recommendations are summarized in Table 4.

**Table 4:** Strengths and limitations of LLMs based on qualitative analysis.

Model	Strength	Limitations
Agentic workflow (HopeAI)	• Comprehensive and up-to-date treatment recommendations • Strong regulatory currency • Best overall performance in complex recommendations	• Incomplete clinical trial data interpretation [Q48] • Inadequate consideration of therapeutic mechanisms [Q8, Q32] • Insufficient supportive care discussion [Q43] • Occasional omission of novel therapies [Q26, 27]
RAG (Myelo)	• Accurate standard treatment protocol recommendations • Clear explanations • Strong in baseline treatment recommendations	• Outdated regulatory information [Q14] • Recommending withdrawn treatments [Q19] • Inadequate treatment specificity [Q20] • Missing key therapeutic options [Q22] • Limited treatment sequencing consideration [Q9]
OpenAI o1-preview	• Clear treatment rationales • Good patient-specific considerations	• Outdated regulatory information [Q2, Q15] • Missing key therapeutic options [Q22, Q47] • Incomplete treatment alternatives discussion • Inappropriate treatment line recommendations [Q34] • Recommending withdrawn medications [Q7]
Gemini 1.5 Pro	• Strong supportive care discussions • Good safety considerations • Detailed complication management	• Overly generic recommendations [Q2, Q46] • Missing critical therapeutic options [Q13, Q14] • Inadequate treatment approach discussions [Q49] • Poor evidence-based support [Q34] • Inaccurate dosing guidance [Q18]
Claude 3.5 Sonnet	• Strong case-specific recommendations • Good comorbidity considerations • Detailed patient-specific factors	• Recommending withdrawn medications [Q6] • Missing critical therapeutic options [Q6, Q49] • Poor treatment setting differentiation [Q47] • Insufficient evidence-based support [Q35] • Poor integration of emerging treatments [Q22]

## Discussion

This study, evaluating the recommendations or responses produced by five LLMs to 50 clinical scenarios related to multiple myeloma, offers several methodological strengths that distinguish it from previous evaluations of AI in medical decision support. First, we implemented a pre-specified and systematic protocol with a custom-developed interface for blinded evaluation, enabling evaluators to assess responses more effectively than traditional spreadsheet-based methods. Second, we established a pre-specified statistical analysis plan to ensure rigorous and unbiased assessment. To our knowledge, this is one of the first large-scale, clinician-blinded evaluations of medical LLM outputs across multiple dimensions, yielding 4,500 rater-response observations.

Although evaluators showed some expected variation across specific scoring dimensions, reflecting the inherent subjectivity of clinical judgment, their consistent overall rankings suggest an ability to distinguish among different AI systems. The agentic workflow achieved superior performance metrics (accuracy 82.0%, relevance 85.3%, comprehensiveness 74.0%), significantly outperforming other approaches. However, the highest rate of ready-to-use recommendations being only 25.3% indicates substantial room for improvement across all platforms. Accordingly, model outputs should be treated as decision support rather than directives, with review by qualified clinicians prior to any real-world use.

Among general-purpose LLMs, OpenAI o1-preview’s performance suggests that architectural advances can enhance domain-specific capabilities. However, the performance gap between general-purpose models and the agentic workflow indicates that specialized approaches may be essential for targeted medical applications. The RAG system (Myelo), while surpassing traditional LLMs in certain metrics, demonstrates that retrieval capabilities alone may inadequately address medical decision support complexities.

Our qualitative analysis highlights a crucial challenge in medical AI models: the gap between information processing and clinical reasoning. While models can effectively process and integrate medical information, they struggle with the complex decision-making required in clinical practice, particularly in rapidly evolving fields like multiple myeloma. This limitation is especially evident in scenarios requiring integration of new therapeutic options, understanding of latest regulatory frameworks, and careful consideration of treatment sequencing. These findings suggest that advancing medical AI systems will require not just improved information processing, but also sophisticated reasoning capabilities that more closely mirror the complex real-life decision-making processes of experienced clinicians. This may be achieved by reasoning processes with deep domain knowledge, rather than relying solely on general language understanding or information retrieval.

Our findings should be interpreted with several limitations. First, the study centers on multiple myeloma; although the protocol is disease-agnostic, generalizability to other cancers remains to be shown and is a priority for future work. Second, scenario selection may introduce bias despite deliberate balancing across diagnostic assessment, NDMM, RRMM, special clinical scenarios, and novel therapeutics/special populations; results should be read together with the reported domain sample sizes.

Future research could extend this comparative study in several important directions. While our current evaluation focused on multiple myeloma and expert clinician assessment, the framework could be easily expanded to other disease areas with complex treatment scheme. Additionally, future evaluations could also incorporate diverse stakeholder perspectives, for example, nurse practitioners, physician assistants, and medical educators could assess clinical utility and training potential. Such expansion would not only test the generalizability of our findings across different medical contexts but also provide insights into developing AI systems that are both clinically accurate and practically useful for all healthcare stakeholders.

## Data Availability

The datasets presented in this study can be found in online repositories. The names of the repository/repositories and accession number(s) can be found at: https://github.com/gnz1013/oncology-llm-evaluation.git.

## Footnotes

1
myeloma.com
