# Supplementary material for: AI for evidence-based treatment recommendation in oncology: a blinded evaluation of large language models and agentic workflows
Source: Front Artif Intell. 2025 Dec 9;8:1683322. doi: 10.3389/frai.2025.1683322 (PMC12722510; doi:10.3389/frai.2025.1683322)
Supplement: Supplementary file 2 [file Data_Sheet_1.docx]

**Supplementary Material for “AI for Evidence-based Treatment Recommendation in Oncology: A Blinded Evaluation of Large Language Models and Agentic Workflows’’**

Guannan Zhai^1^, Merav Bar^2^, Andrew J. Cowan^3,4^, Samuel Rubinstein^5^, Qian Shi^6^, Ningjie Zhang^7^, En Xie^8^, Will Ma^8^

^1^ Department of Statistics, George Washington University, Washington, DC

^2^ Bristol Myers Squibb, Summit, NJ

^3^ Division of Hematology-Oncology, University of Washington, Seattle, WA

^4^ Clinical Research Division, Fred Hutch Cancer Center, Seattle, WA

^5^ Division of Hematology, University of North Carolina, Chapel Hill, NC

^6^ Department of Quantitative Health Sciences, Mayo Clinic, Rochester, MN

^7^ Department of Bioinformatics and Biostatistics, Shanghai Jiao Tong University, Shanghai, CN

^8^ Hope AI, Inc, Princeton, NJ

Corresponding Author:

Will Ma

HopeAI, Inc., 103 Carnegie Center Dr, Suite 300, Princeton, NJ 08540

Email: will@hopeai.co

**Summary**

This supplementary material contains three sections. Section [A](#_A._Study_Protocol) details a study protocol for evaluating Large Language Models (LLMs) in clinical decision support through a blinded, comparative assessment of three types of LLMs: general-purpose LLMs, Retrieval Augmented Generation (RAG), and an agentic workflow. The protocol includes standardized scoring guidelines and statistical analysis methods for comparing these implementations' performance. Section [B](#_B._Full_List) provides a full list of 50 clinical scenarios used in the evaluation, covering diverse medical decision-making situations. Section [C](#_C._Additional_Results) reports additional results, including a domain-level performance summary.

# **A. Study Protocol**

1. **Introduction**

**1.1 Background**

Evidence-based medicine (EBM) is considered as the cornerstone of clinical practice, guiding healthcare professionals in making informed decisions about patient care. However, studies have shown that approximately 80% of treatment decisions are not based on the latest evidence, highlighting a significant disconnect between current medical knowledge and its application in clinical settings. This discrepancy not only undermines the quality of patient care but also potentially exposes patients to suboptimal or even harmful treatments.

The advent of Large Language Models (LLMs) has sparked interest in their potential to bridge this gap by providing evidence-based treatment recommendations. These AI-powered systems can process vast amounts of medical literature and data, offering the promise of up-to-date, evidence-based guidance at the point of care. However, recent studies have revealed limitations in the reliability of general-purpose LLMs for medical applications [1,2,3]. For instance, research has shown that approximately one-third of cancer treatment recommendations made by ChatGPT contain incorrect information that could be potentially harmful to patients [2].

To address these limitations, new technologies have emerged. Retrieval Augmented Generation (RAG) enhances LLMs by enabling them to provide answers based on specific content rather than relying solely on pre-trained knowledge. Additionally, Agentic Workflows (Agent) have been developed to handle more complex queries, including the synthesis of evidence with statistical analyses. These approaches aim to improve the accuracy and reliability of AI-generated medical recommendations.

Furthermore, the role of human curation in ensuring the reliability of LLM-generated answers cannot be overstated. By combining the processing power of AI with human expertise, it may be possible to create systems that offer more trustworthy and clinically relevant guidance.

**1.2 Rationale for the Study**

Given the potential impact of AI on healthcare decision-making and the varying approaches to improving LLM performance in medical contexts, there is a critical need for rigorous evaluation of these technologies. This study aims to contribute valuable insights to the ongoing dialogue about the role of artificial intelligence in healthcare by comparing the performance of general-purpose LLMs， Retrieval-Augmented Generation systems, and Agentic Workflows in providing evidence-based treatment recommendations.

By assessing platforms such as ChatGPT o1-preview model, Claude 3.5 Sonnet, Gemini 1.5 Pro, Myelo and HopeAI through a double-blinded evaluation process, we seek to objectively measure their capabilities, limitations, and potential for improving evidence-based practice in medicine. The findings of this study will not only inform the development and refinement of AI technologies for healthcare applications but also guide healthcare professionals and policymakers in understanding how these tools can be effectively and safely integrated into clinical practice.

The significance of this study lies in its potential to:

1. Inform the medical community about the capabilities and limitations of LLMs in handling complex, disease-specific clinical inquiries.
2. Highlight the potential of LLMs as supportive tools for healthcare providers in patient management.
3. Identify areas where LLMs perform well or need improvement in interpreting specialized medical knowledge, informing future model developments.
4. Assess how well LLMs keep pace with the rapidly evolving research and treatment in specific medical indications.
5. Evaluate the contribution of LLMs to enhancing clinical decision-making in hematology-oncology.

**2. Objective**

**2.1 Primary Objective**

To compare the accuracy, relevance, completeness, and clarity of general-purpose large language models (LLMs), Retrieval-Augmented Generation (RAG) and Agentic Workflow (Agent) in evidence-based treatment recommendations.

**2.2 Secondary Objectives**

Consistency: Measure the consistency of LLMs for different variations of a question.

Benefit: Evaluate the potential benefit of using these LLMs on the efficiency and quality of patient care.

Risk: Identify potential risks of using LLMs in treatment decisions of patient care.

**3. Study Design and Rationale**

**3.1 Study Design**

The double-blinded evaluation of LLMs in treatment recommendation was sponsored by HopeAI in collaboration with researchers from Mayo Clinic, Fred Hutch Cancer Center, and Bristol Myer Squibb. Three general purpose large language models - ChatGPT o1-preview model, Claude 3.5 Sonnet, Gemini 1.5 Pro,one RAG-based LLM - Myelo and one agentic workflow-based LLM - HopeAI were included in the study.


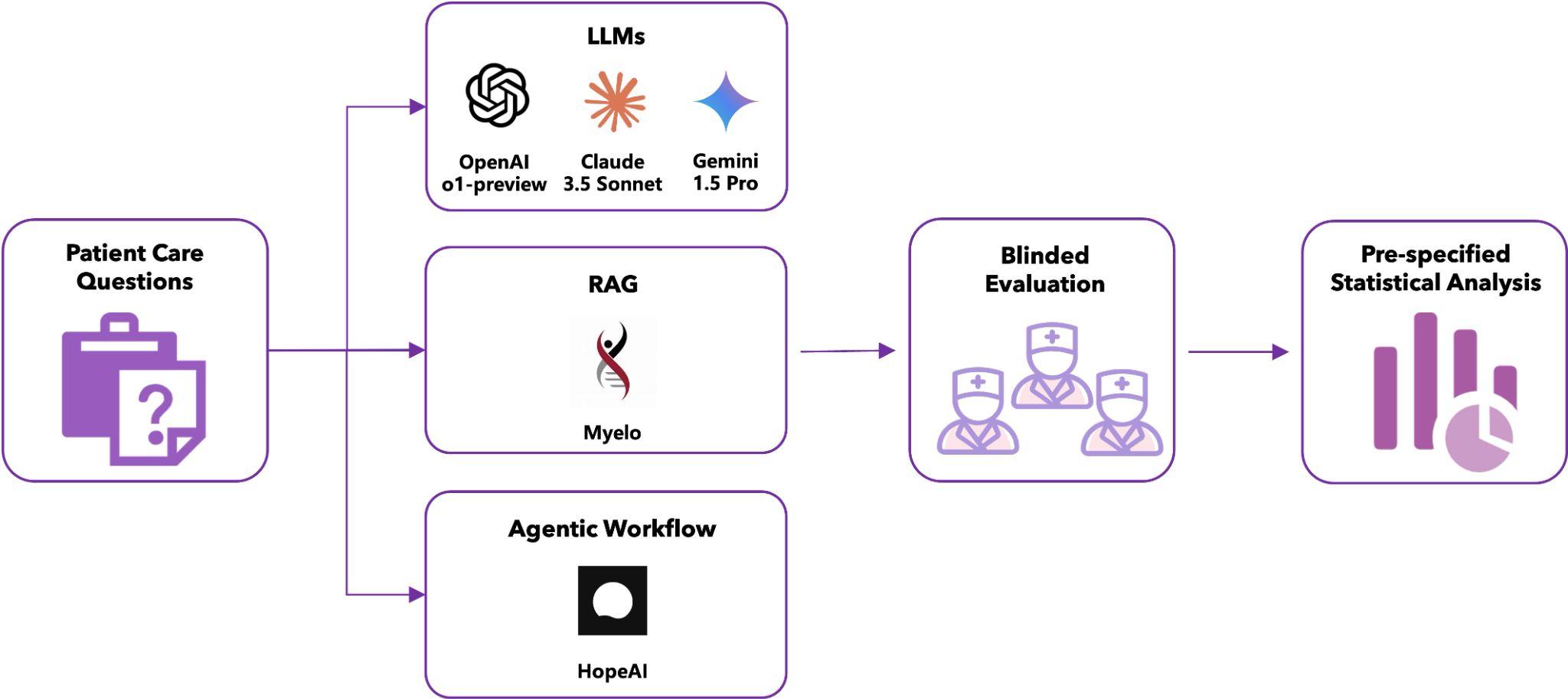


**3.1.1 Question Preparation**

A panel of hematologist-oncologists specializing in clinical diagnosis and treatment will be engaged to develop a set of at least 50 clinical questions covering various aspects of the disease. These questions will encompass topics such as diagnosis, staging, treatment options, prognosis, and management of complications. The questions will be reviewed to ensure clarity, clinical relevance, and appropriate difficulty level. This comprehensive set of questions will allow for a thorough evaluation of the LLMs' knowledge and reasoning capabilities.

All questions will be summarized in a table in a csv file containing the columns ‘Number’ and ‘Question’.

**3.1.2 Comparative Study System**

The Comparative Study System is a platform developed to allow physicians to upload questions and evaluate the responses of Language Learning Models in a blinded manner. This platform provides a structured workflow for creating, executing, and analyzing responses to patient care questions. Key features include a simple step to upload question files, a blinded process to ensure unbiased evaluations, a secure interface for uploading and managing question files, and an automated system for distributing questions to LLMs for answer generation.

The platform facilitates a thorough assessment of LLM performance across five dimensions. The scoring interface is equipped with tools for detailed evaluation, and results are presented in a clear, visual format, including tables and stacked bar charts. This system is a valuable resource for professionals in the field of language model research and development, offering a comprehensive solution for comparative analysis and performance benchmarking.

**3.1.2.1 Instructions for Using the System**

1. On the system login homepage, historical projects are displayed. To create a new comparative test task, click on the “+ New Project” button.


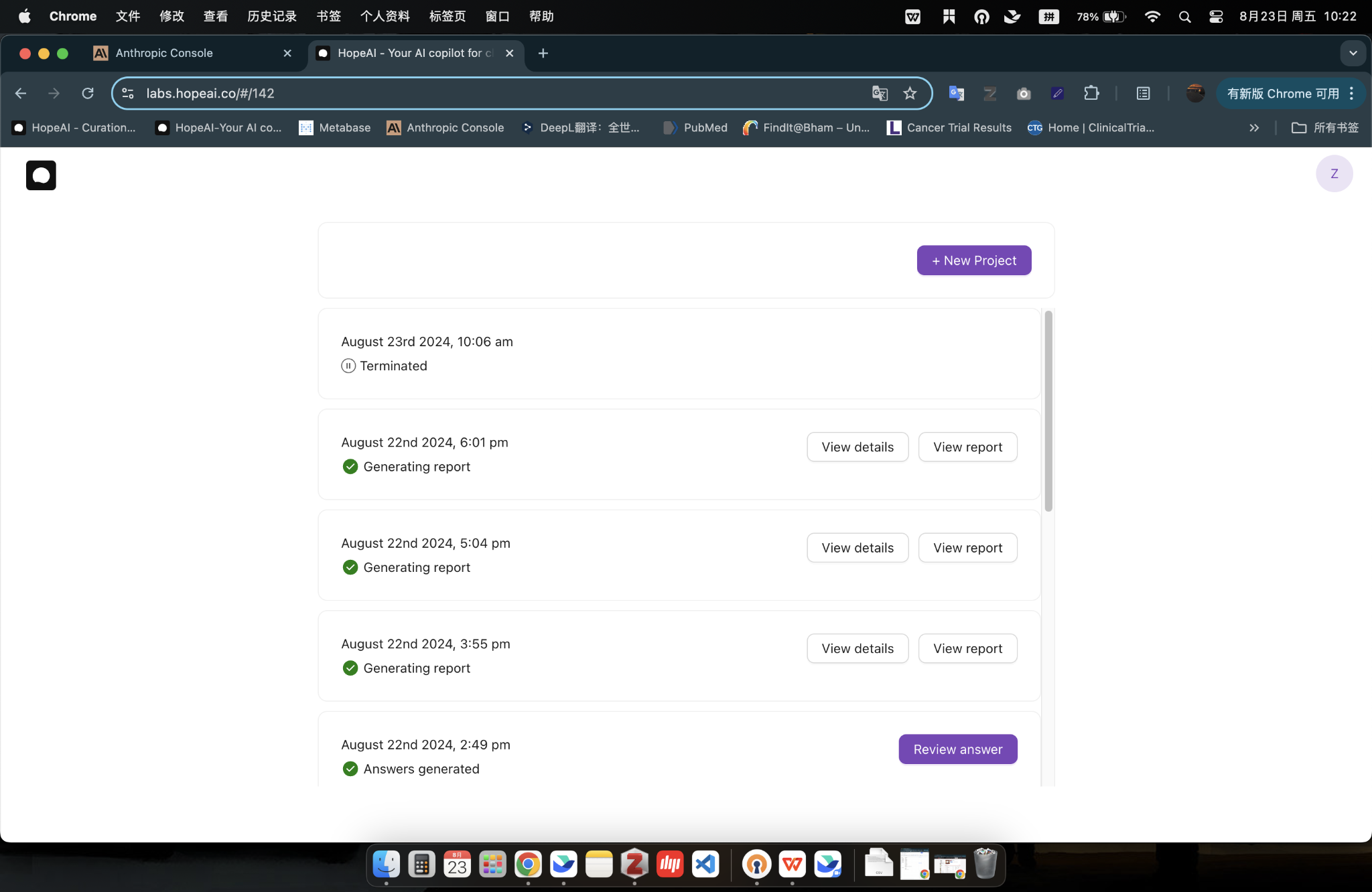


1. A window will pop up, allowing you to upload the question file for the comparative test by dragging or clicking. Only CSV format is supported for upload. The CSV file must contain two columns: “Number” and “Question”.


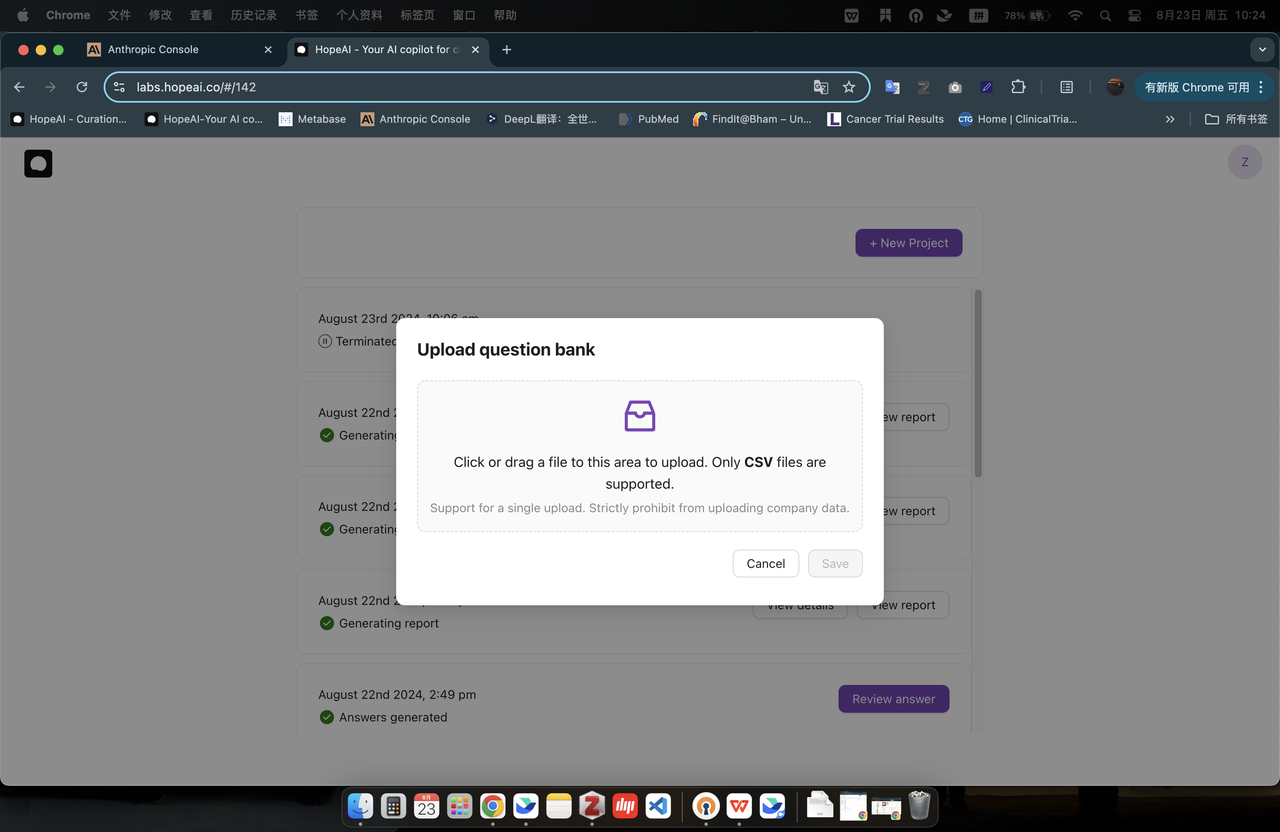


1. After uploading the question table, click “save” to complete the project setup.


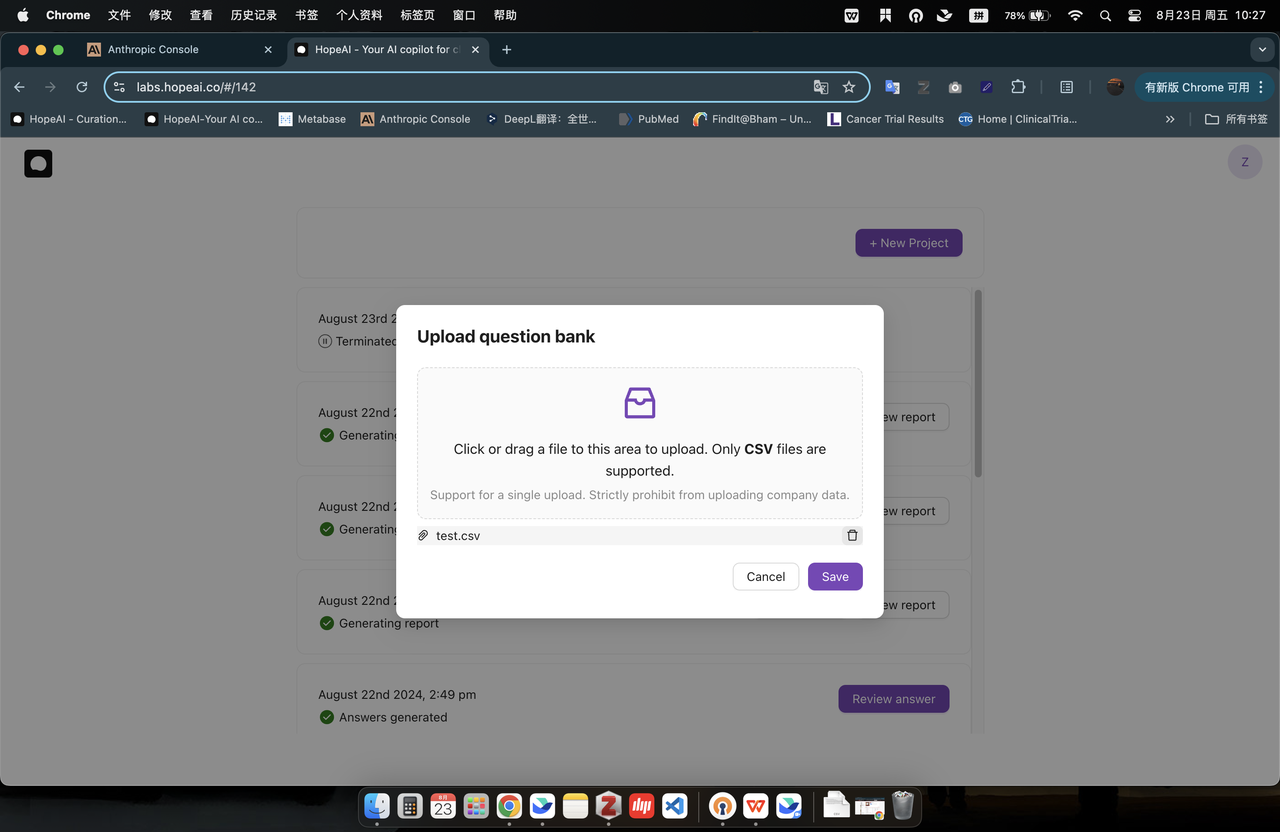


1. The system will automatically distribute the questions to various LLMs for answer generation.


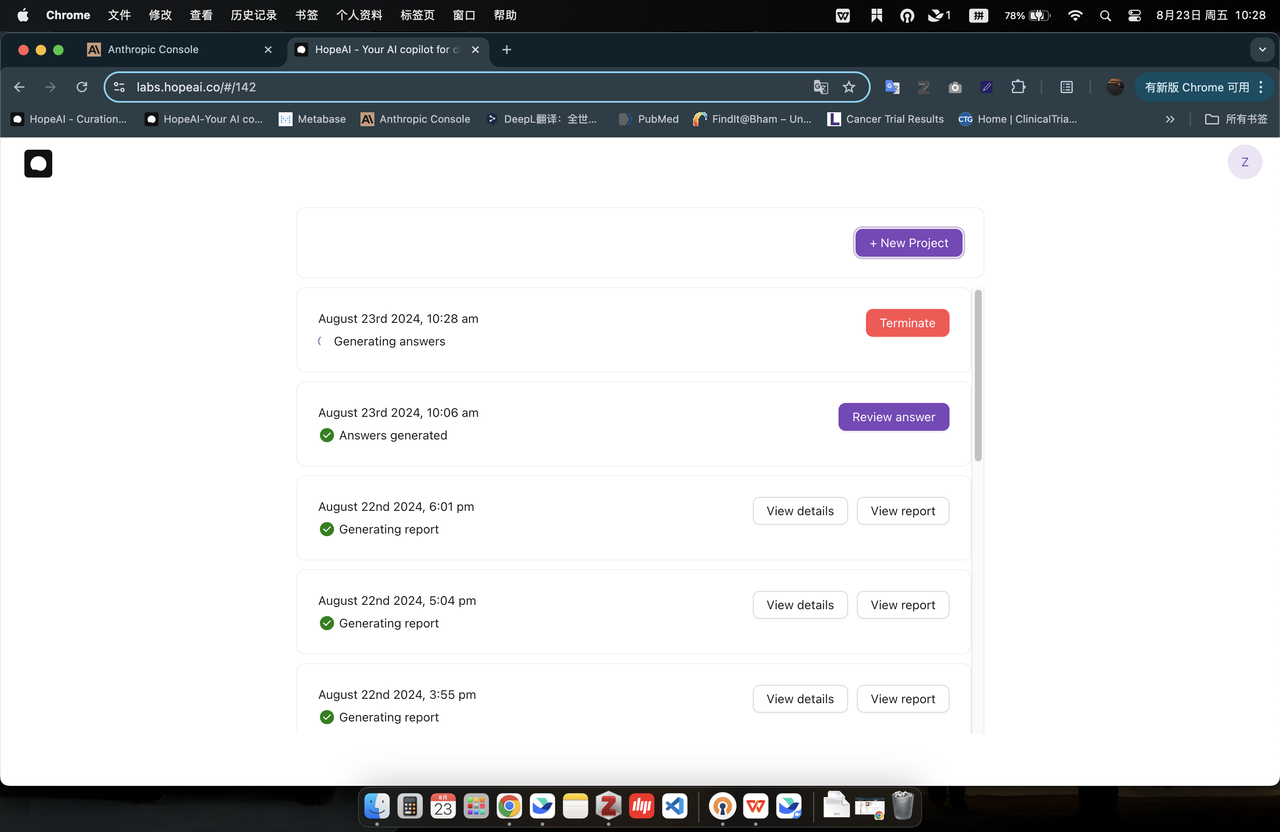


1. Once answer generation is complete, click “Review answer” to view the different answers generated by the LLMs.


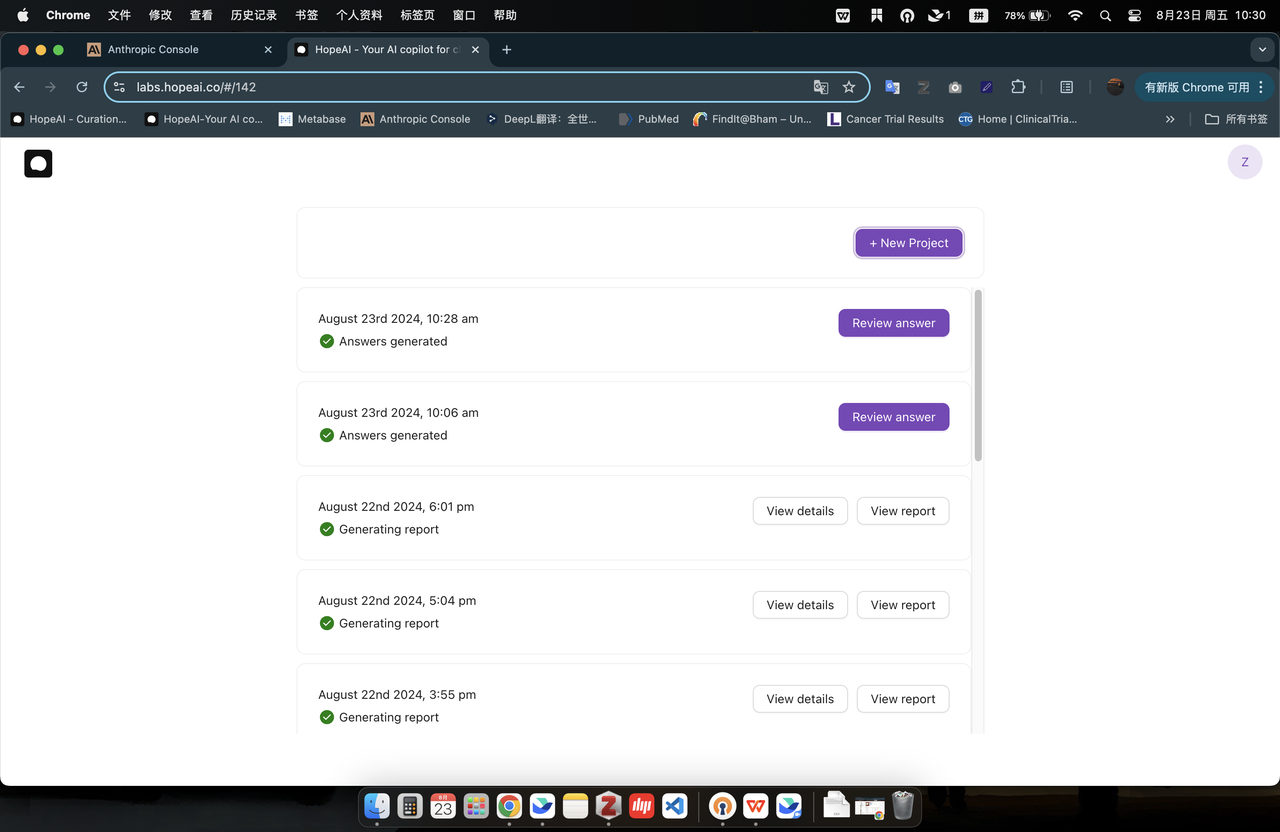


1. The scoring interface appears as follows. The leftmost column represents the question number, the middle is the answer display area, with tabs for different LLMs (Model-1, Model-2, Model-3, Model-4, Model-5). These are random aliases for different LLMs, and each project will reshuffle the aliases to avoid subjective bias. The rightmost column contains questions assessing five main dimensions of the LLM answers. Question 6 (optional) allows for evaluations on additional dimensions.


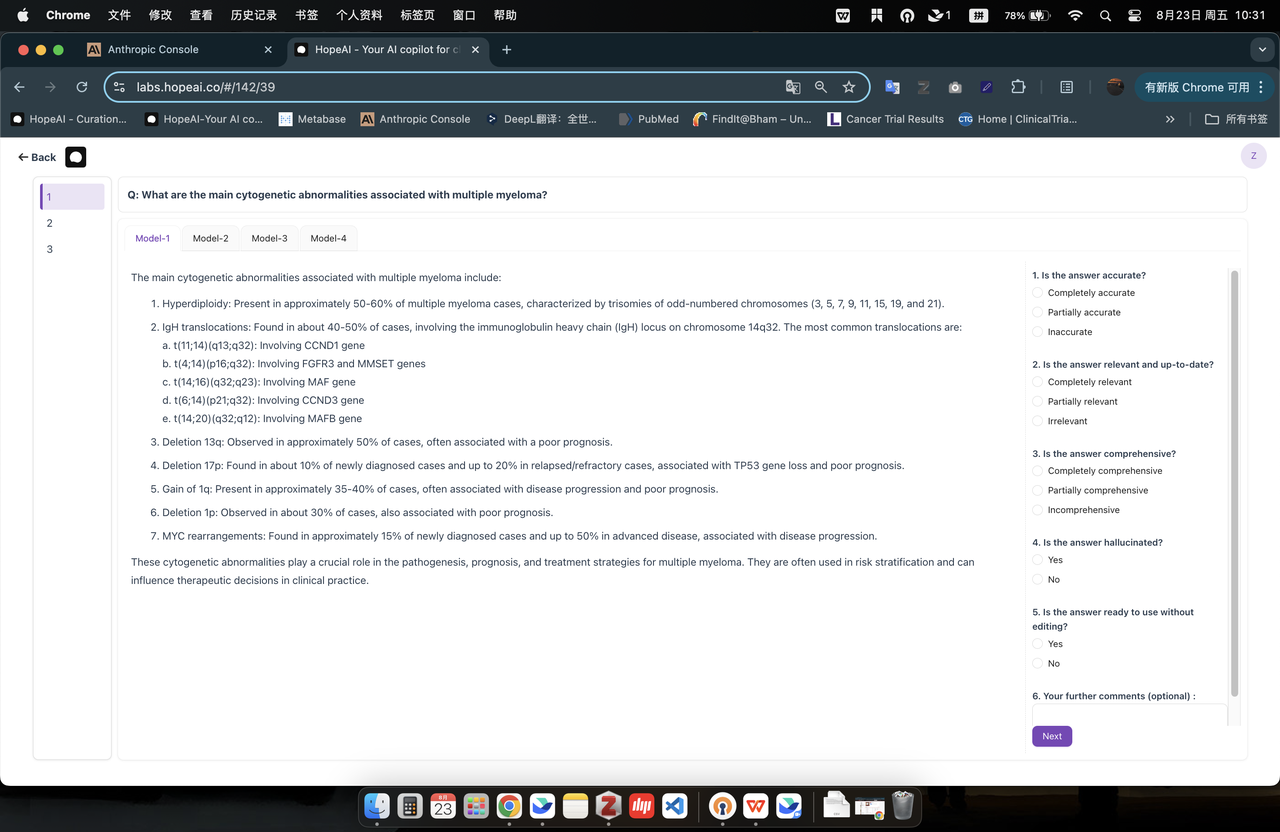


1. Click “Next” to switch between different LLMs and questions. Note that you cannot switch if there are blank questions on the current page.


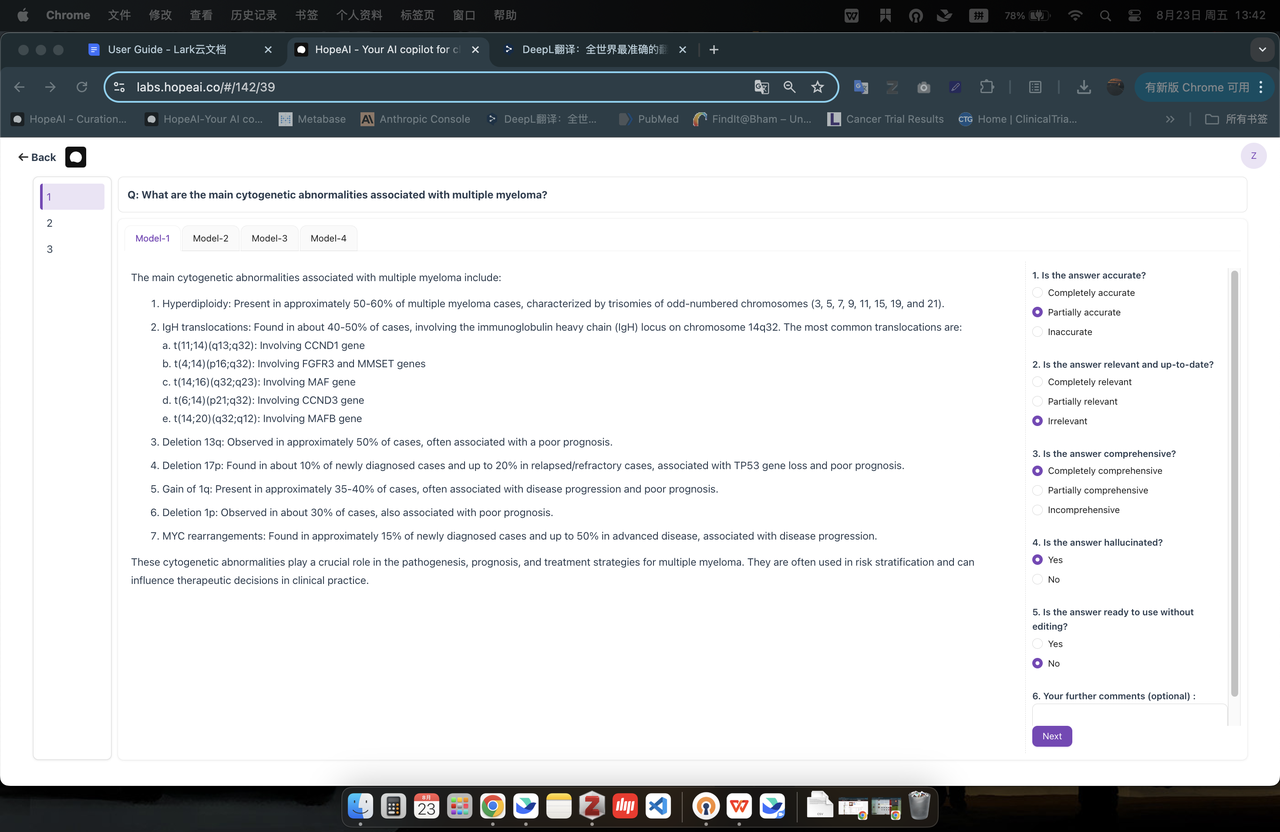


1. A “√” mark will appear next to the question number once the assessment for all LLMs is complete. If there is no mark, it indicates that there are still questions left to be answered. The “Submit” button will only appear when all questions for all LLMs are completed.


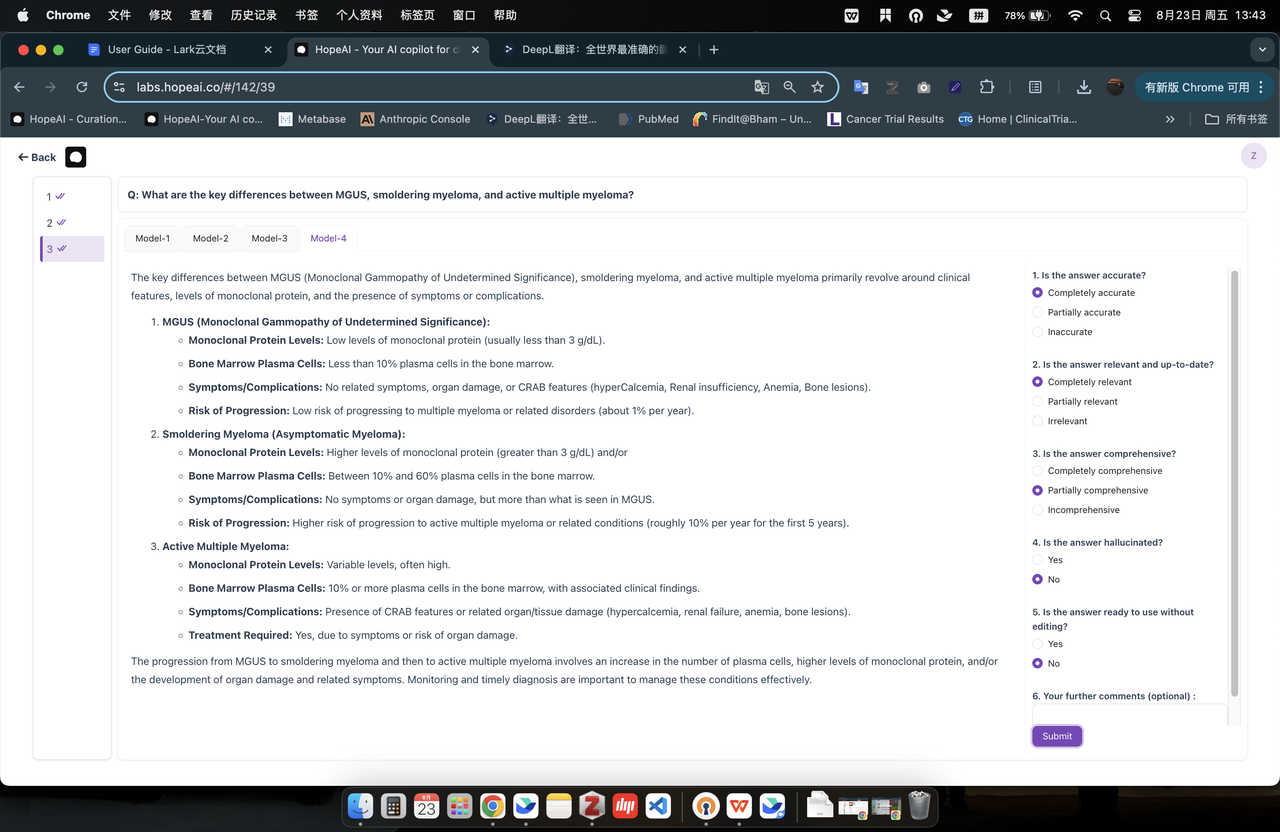


1. After clicking “Submit”, a pop-up window will appear, requiring confirmation. Click “Confirm” to officially complete the scoring.


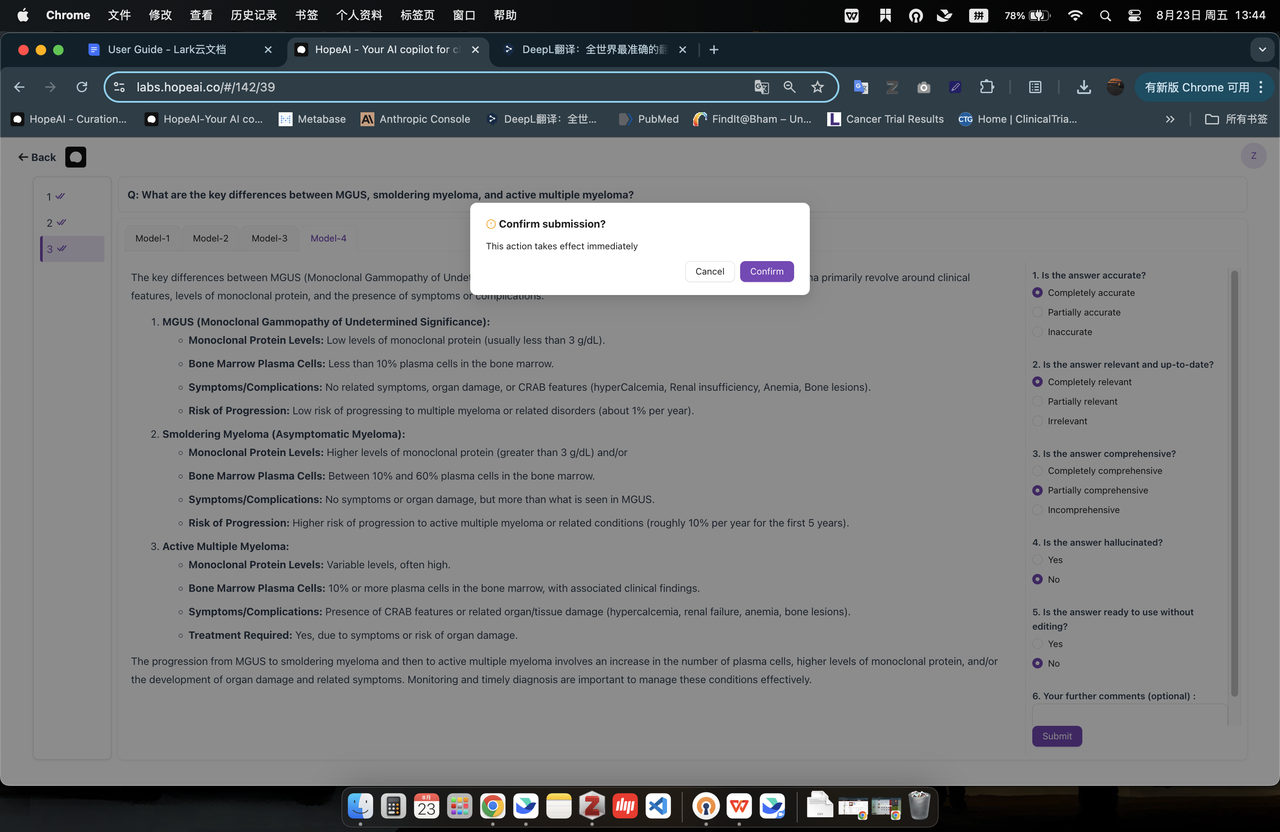


1. Upon successful submission, the system will automatically return to the project interface. Click “View details” to review the submitted scores, but no changes can be made.


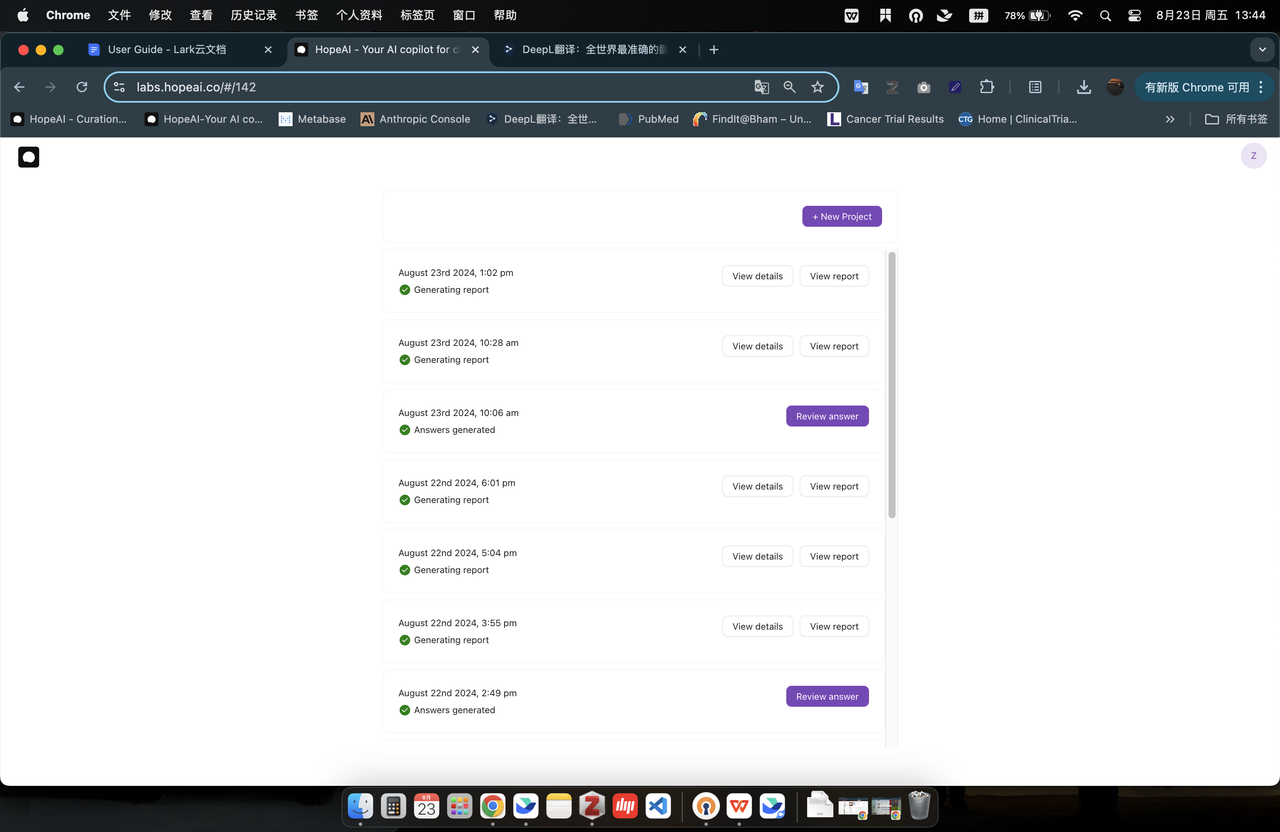


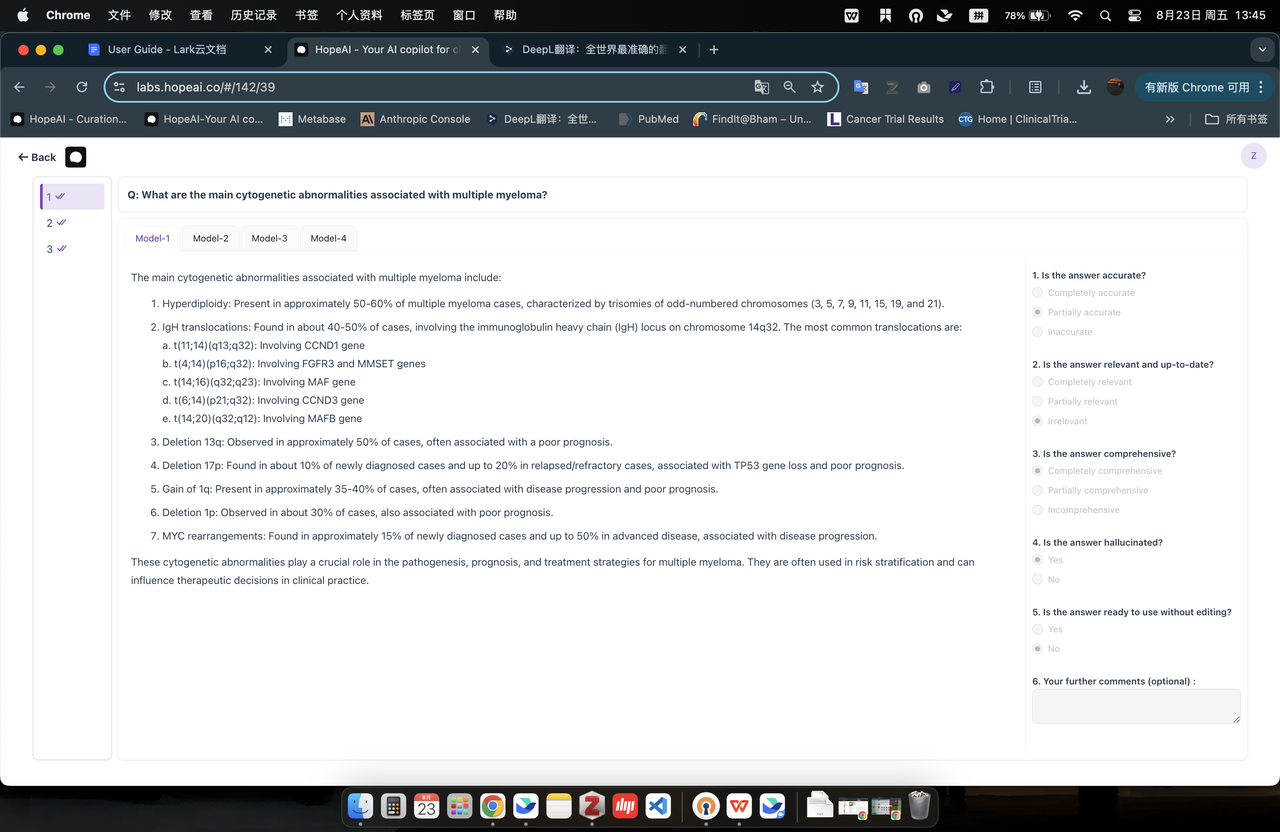


1. On the project interface, click “View report” to reveal the post-unblinding evaluation comparison results. The actual LLMs corresponding to the Model numbers will be announced. The scoring statistics are visualized through tables and stacked bar charts, showing the proportion of options for each LLM across different dimensions.


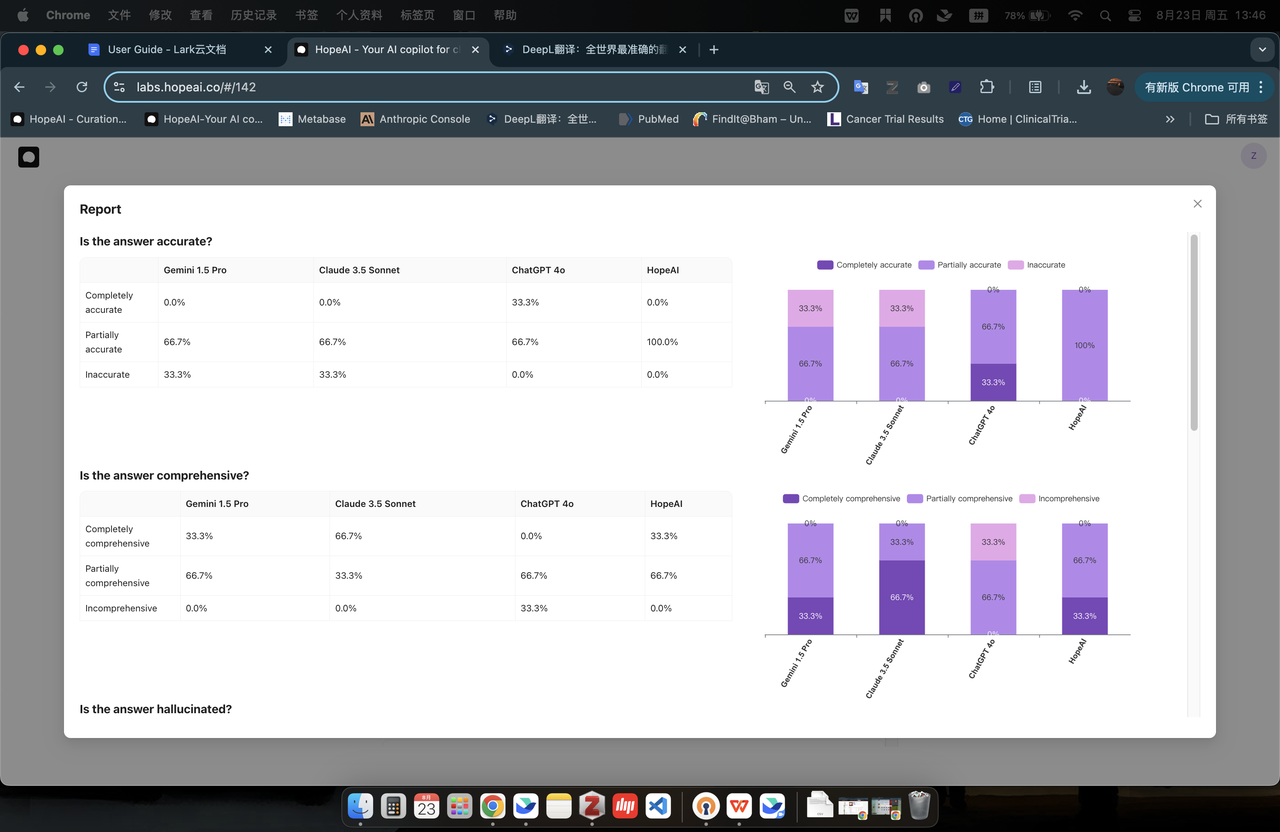


**3.1.3 Blinded Evaluation**

The evaluation process will be carried out by a panel of 3 independent hematologist-oncologists. These experts will be selected based on their extensive experience and expertise in various fields of medicine. They will have no affiliations with the development or promotion of any of the LLMs being tested to ensure impartiality. Each evaluator will independently review and score the responses using the predefined scoring criteria. To maintain the integrity of the study, all evaluators will undergo training to familiarize themselves with the scoring guidelines and ensure consistency in their evaluations. The evaluators will be instructed not to discuss their assessments with each other until after the evaluation process is complete to maintain the integrity of the blinded review. The diversity in their medical backgrounds will also provide a robust and multifaceted assessment of the LLMs, reflecting different perspectives and areas of expertise within clinical practice.

**3.1.4 Result Analysis**

After all evaluations are complete, the platform system will reveal the LLM identities linked to each answer. The scores will then be matched to their respective systems. Statistical analyses, including average scores calculation, analysis of variance (ANOVA), and rank-based tests will be conducted to identify significant performance differences across the models. Detailed statistical analysis procedures and methods can be found in the attached Statistical Analysis Plan (SAP).

This updated protocol aims to provide a robust and unbiased assessment of the LLMs' performance in addressing specialized clinical questions, ensuring complete confidentiality and integrity throughout the process.

**3.2 Rationale of the Study Design**

The experimental design described in the protocol is structured to ensure an objective and effective evaluation of language model performance in a clinical setting. Here's a reorganized explanation of its rationality.

Blinding is integral to the design, where answers generated by the language models are presented without identifiers, ensuring judgments are based solely on answer quality. This prevents any bias that might arise from evaluators' preconceptions about the capabilities of specific models. Additionally, the confidentiality of the test questions safeguards the integrity of the experiment, ensuring that no external influences sway the outcomes.

Utilizing a public network platform to connect the APIs of all models involved ensures a controlled environment where operational parameters are consistent across all entries. This standardization is crucial for fair comparison, minimizing external variables that could influence the performance assessments beyond the inherent capabilities of the models.

Each question is processed by multiple models, generating a diverse array of responses. This approach not only enhances the comparative analysis but also mirrors realistic scenarios where different models might be employed to provide solutions. The independent scoring by experienced hematologist-oncologists, based on a detailed rubric, further reinforces the reliability of the evaluation process.

Post-evaluation, the unblinding of model identities allows for a transparent linkage between the models and their corresponding scores. The use of statistical analyses, including ANOVA and post-hoc tests, facilitates a thorough examination of the data to discern statistically significant differences in model performance. This step is essential for identifying both the strengths and limitations of each model, guiding potential improvements and applications in clinical practice.

This methodical approach to the evaluation of language models in a clinical context ensures that the study's findings are both reliable and applicable, providing valuable insights into the potential utility of these models in real-world medical settings.

**4. Large Language Models (LLMs)**

This study compares the performance of three categories of AI models in answering clinical questions: General-Purpose LLMs, RAGs and Agentic Workflows. Each category represents a different approach to artificial intelligence and natural language processing, with unique characteristics and potential advantages in medical applications.

**4.1 General-Purpose Large Language Models (LLMs)**

Large Language Models are AI systems trained on vast amounts of text data to understand and generate human-like text. They can perform a wide range of language tasks without being specifically designed for any particular domain.


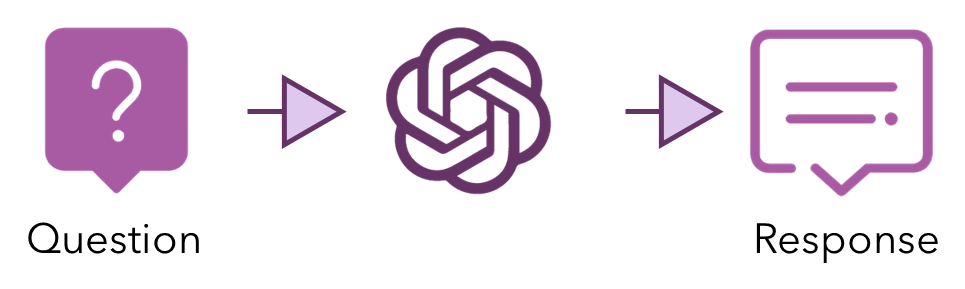


**4.1.1 ChatGPT o1-preview model**

The ChatGPT o1-preview model represents a significant advancement in AI reasoning capabilities, designed for solving complex problems in science, coding, and math. This model spends more time thinking through issues before responding, akin to a human’s thought process. It demonstrates remarkable performance on challenging benchmark tasks, resembling PhD students in physics, chemistry, and biology, and excelling in math and coding contests. Although it lacks certain features of ChatGPT, such as web browsing and file uploads, the o1 preview model marks a new level of AI capability for intricate reasoning tasks. Additionally, it adheres to enhanced safety and alignment guidelines, showcasing improved jailbreaking resistance. This model is particularly beneficial for professionals in healthcare, physics, and software development, facilitating data annotation, complex formula generation, and multi-step workflow execution.

**4.1.2 Claude 3.5 Sonnet**

Claude 3.5 Sonnet, the latest release in the Claude model family, sets new industry standards for intelligence, outperforming previous models and competitors on a variety of evaluations. Operating at twice the speed of Claude 3 Opus, it excels in graduate-level reasoning, undergraduate knowledge, and coding proficiency, with notable improvements in understanding nuance, humor, and complex instructions. Available for free on Claude.ai and the Claude iOS app, with higher rate limits for subscribers, Claude 3.5 Sonnet is cost-effective and ideal for complex tasks like customer support and multi-step workflows. It also showcases advanced vision capabilities, excelling in visual reasoning tasks and text transcription from images. The model is committed to safety and privacy, having undergone rigorous testing and training to reduce misuse, and it maintains ASL-2 safety rating. Claude 3.5 Sonnet paves the way for future releases and features, aiming to enhance user experience and support a wide range of business applications.

**4.1.3 Gemini 1.5 Pro**

Gmini 1.5 Pro is the latest iteration of Google’s Gemini family of multimodal language models. Building upon the previous Gemini 1.0 models, 1.5 Pro boasts a significantly expanded context window, allowing it to process and reason over information from up to 10 million tokens, including extensive text, video, and audio content. This unprecedented capability enables groundbreaking applications such as in-context language learning and comprehensive analysis of complex documents and videos. Despite its impressive gains in long-context understanding, 1.5 Pro maintains and even surpasses the performance of its predecessor across a wide range of benchmarks, including math, science, reasoning, coding, and multilingual tasks.

**4.2 Retrieval Augmented Generation (RAG)**

RAG systems combine the capabilities of large language models with the ability to retrieve and incorporate external information from a curated knowledge base. This approach allows for more up-to-date and verifiable responses.


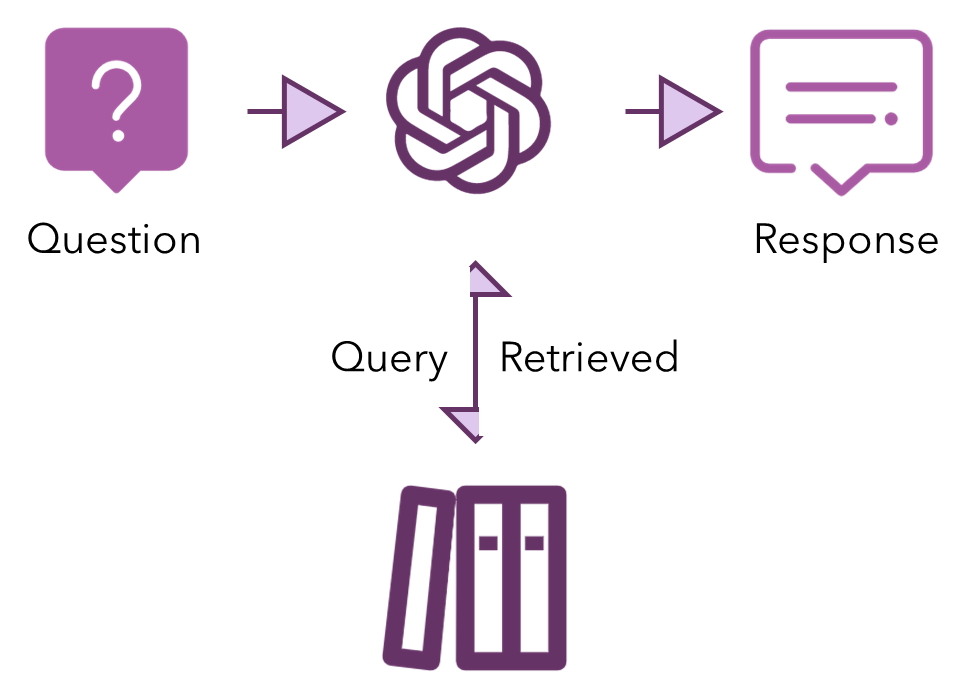


**4.2.1 Myelo**

Myelo is an indication-specific chatbot developed by International Myeloma Foundation (myeloma.com) in collaboration with ZS Associates and AWS. It is designed to provide compassionate support for multiple myeloma patients, caregivers and health professionals [4]. Equipped with extensive knowledge about symptoms, diagnosis, treatment options, clinical trials, and management strategies, Myelo offers compassionate and caring responses to frequently asked questions.

**4.3 Agentic Workflows (Agent)**

Agentic workflows involve AI systems that can autonomously plan and execute a series of actions to achieve a specific goal. These systems often combine language models with task-specific modules and decision-making capabilities.


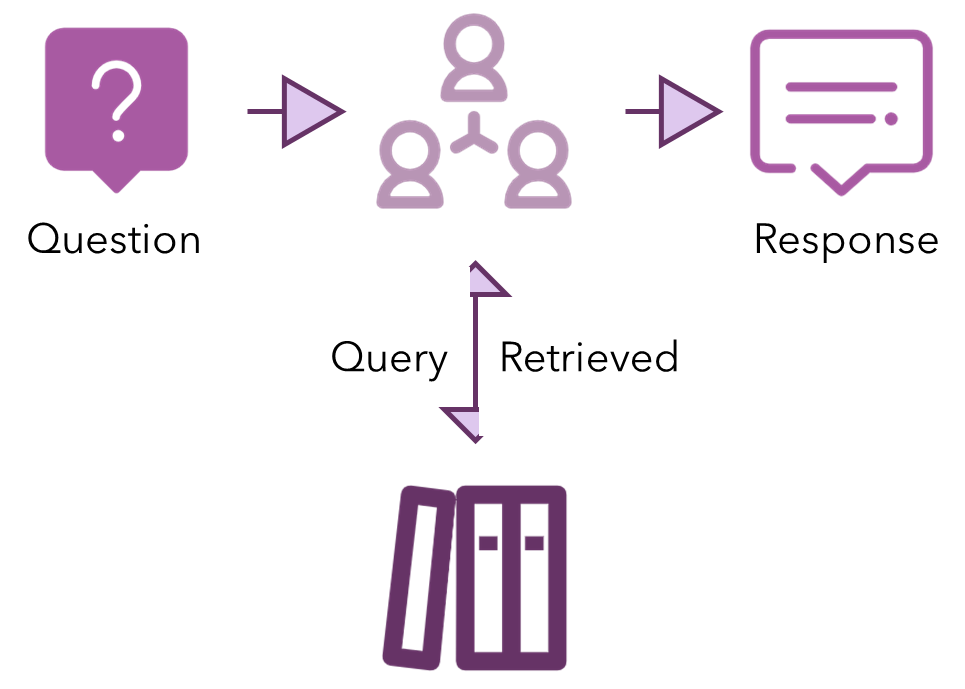


**4.3.1 HopeAI**

HopeAI is an AI copilot (mm.hopeai.co) designed to bring comprehensive and up-to-date clinical evidence to the fingertips of clinical teams for evidence-based trial design and treatment decisions. It utilizes a combination of AI technology and human curation to provide reliable clinical insights that bridge the gap between clinical literature and clinical insights.

The process begins with data extraction from clinical literature, followed by standardization and live meta-analysis to generate analysis data. This expert-curated data is then used to derive clinical insights for clinical trial optimization and precision medicine.

**5. Scoring Guideline**

**5.1 Introduction**

The primary objective of this study is to compare the performance of three different kinds of LLMs in providing evidence-based treatment recommendations: general purpose LLMs, RAG and Agent. We aim to evaluate how well each approach can provide accurate, comprehensive, and actionable treatment recommendations based on current clinical evidence.

This scoring system is designed to assess multiple aspects of the recommendations provided by each approach, including accuracy, relevance, comprehensiveness, non-hallucination and usability. By systematically evaluating these aspects, we aim to gain insights into the strengths and limitations of each approach in the context of evidence-based medicine. This will help us understand the potential applications and risks associated with using these technologies in clinical decision support.

The following scoring guidelines will be used to assess the outputs from each approach (general purpose LLMs, RAG and Agent) across multiple medical scenarios. Evaluators should carefully consider each response in its entirety before assigning scores for individual criteria.

**5.2 Scoring questions and definitions**

1. Is the answer accurate?

Value set:

- Completely accurate
- Mostly accurate
- Partially accurate
- Inaccurate

Reflect on the reliability of each model in providing accurate information without causing harm. Consider the implications of inaccuracies in clinical decision-making scenarios.

1. Is the answer relevant and up-to-date?

Value set:

- Completely relevant
- Mostly relevant
- Partially relevant
- Irrelevant

Reflect on how well each model delivers information that is pertinent to the clinical problem and avoids misleading conclusions. Consider the impact of relevance on the usefulness of model outputs.

1. Is the answer comprehensive?

Value set:

- Completely comprehensive
- Mostly comprehensive
- Partially comprehensive
- Incomprehensive

Reflect on which models consistently provide thorough and detailed answers that cover all relevant aspects of the clinical problem. Identify areas where models may lack depth of coverage.

1. Is the answer hallucinated?

Value set:

- Yes
- No

Reflect on the reliability and coherence of each model’s outputs. Consider how hallucinated responses impact the overall credibility and trustworthiness of the models.

1. Is the answer ready to use without editing?

Value set:

- Yes
- No

Reflect on the appropriateness of language and tone used by each model, ensuring they do not promote unethical content or advice. Consider user feedback on the clarity and usefulness of model outputs.

1. Your further comments **(optional)**:

**6. Statistical Analysis Plan**

This Statistical Analysis Plan (SAP) outlines the comprehensive approach for evaluating the performance of all 5 AI models on clinical diagnosis and treatment recommendation questions. The models under evaluation include ChatGPT o1-preview model, Claude 3.5 Sonnet, Gemini 1.5 Pro, and HopeAI. The assessment will be based on five key criteria: Accuracy, Relevance, Comprehensiveness, Non-hallucination, and Usability. A total of 50 questions will be used for this evaluation.

**6.1 Primary endpoints**

6.1.1 Accuracy

6.1.1.1 Definition

Accuracy is defined as the degree to which the AI model's response aligns with established medical knowledge and best practices. It will be evaluated on a four-point scale: Completely accurate, Mostly accurate, Partially accurate, and Inaccurate.

6.1.1.2 Statistical Methods

The primary analysis for accuracy will focus on the proportion of answers from each model that fall into the different accuracy levels. The response categories will be clearly defined and explained according to the scoring system. For each model, 95% exact confidence intervals (CI) will be calculated to provide a measure of precision for the estimated proportions.

To detect differences in accuracy across the models, a stratified Cochran-Mantel-Haenszel (CMH) test will be employed. This test will account for potential confounding factors and allow for a more nuanced comparison between the models. The odds ratios estimated by the CMH test, along with their 95% confidence intervals and p-values, will be reported for pairwise comparisons between different models.

Stratification factors will be considered in the analysis, grouping the models into three categories: General-Purpose LLMs (ChatGPT o1-preview model, Claude 3.5 Sonnet, Gemini 1.5 Pro), RAG (Myelo) and Agent (HopeAI). This stratification will allow for more meaningful comparisons between different types of AI models.

6.1.2 Relevance

6.1.2.1 Definition

Relevance assesses how well the model's response addresses the specific question or clinical scenario presented. It will be evaluated on a three-point scale: Completely relevant, Mostly relevant, Partially relevant, and Irrelevant.

6.1.2.2 Statistical Methods

Similar statistical methods will be applied as described in Section 6.1.1.2 for accuracy analysis. The proportion of answers in each relevance category will be calculated for each model, with 95% exact confidence intervals provided. The stratified CMH test will be used to detect differences in relevance across models, with odds ratios, confidence intervals, and p-values reported for pairwise comparisons. The same stratification factors will be applied to ensure consistency in the analysis.

6.1.3 Comprehensiveness

6.1.3.1 Definition

Comprehensiveness evaluates the completeness and depth of the model's response, assessing whether it covers all necessary aspects of the clinical question. It will be rated as Completely comprehensive, Mostly comprehensive, Partially comprehensive, or Incomprehensive.

6.1.3.2 Statistical Methods

Similar statistical methods will be applied as described in Section 6.1.1.2 for accuracy analysis. Proportions of responses in each category will be calculated, along with 95% confidence intervals. The stratified CMH test will be employed to compare comprehensiveness across models, with the same reporting of odds ratios, confidence intervals, and p-values. Stratification factors will remain consistent with previous analyses.

6.1.4 Hallucination

6.1.4.1 Definition

Non-hallucination assesses whether the model's response contains fabricated or false information not supported by established medical knowledge. It will be evaluated as a binary outcome: Yes (hallucination present) or No (no hallucination present).

6.1.4.2 Statistical Methods

For Hallucination, the proportion of responses without hallucinations will be calculated for each model, accompanied by 95% confidence intervals. Given the binary nature of this outcome, logistic regression analysis will be used to compare the odds of non-hallucination across models, adjusting for the stratification factors. Odds ratios, confidence intervals, and p-values will be reported for these comparisons.

6.1.5 Usability

6.1.5.1 Definition

Usability evaluates whether the model's response is ready to use without editing, considering factors such as clarity, conciseness, and practical applicability. It will be assessed as a binary outcome: Yes (ready to use) or No (requires editing).

6.1.5.2 Statistical Methods

Similar statistical methods will be applied as described in Section 6.1.4.2 for non-hallucination analysis., using logistic regression to compare the odds of usability across models while adjusting for stratification factors. Proportions of usable responses, along with 95% confidence intervals, will be reported for each model.

**6.2 Secondary endpoints**

6.2.1 The proportion of correct answers

To further analyze the performance of the models, chi-square tests will be conducted to examine differences in the proportion of correct answers (defined as completely accurate and completely relevant) among the models. This analysis will provide insights into the overall correctness of responses across all evaluated criteria.

6.2.2 Significance of differences

Two levels of comparison will be performed to assess the significance of differences in performance:

6.2.2.1 Among all LLMs

An analysis of variance (ANOVA) will be conducted to compare the overall performance across all models simultaneously. If the assumptions for ANOVA are not met, a Kruskal-Wallis test will be used as a non-parametric alternative.

6.2.2.2 Between specific LLM categories

Pairwise t-tests will be performed to compare the performance between the best-performing General-Purpose LLM, RAGs and Agentic Workflows. These comparisons will help identify any significant advantages of specialized LLMs over general-purpose models.

**6.3 Ethical Considerations**

The statistical analysis plan acknowledges the importance of ethical considerations in evaluating AI models for clinical applications. All evaluations will strictly adhere to ethical guidelines, particularly concerning the handling of patient data and the provision of clinical advice. The appropriateness of language and advice provided by each model will be carefully assessed as part of the evaluation process. Any potential ethical concerns identified during the analysis will be documented and reported alongside the performance metrics.

**6.4 Limitations**

The SAP recognizes potential limitations that may affect the interpretation of results. These include possible biases in the dataset used for evaluation or in the training data of the models. The analysis will attempt to identify and quantify any such biases to provide context for the findings. Additionally, constraints in model capabilities or data availability that may impact the comprehensiveness of the evaluation will be acknowledged and discussed in the final report. The stratification approach in the analysis aims to mitigate some of these limitations by allowing for more nuanced comparisons between different types of AI models.

# **B. Full List of 50 Clinical Scenarios**

| **Number** | **Clinical Scenarios** |
| --- | --- |
| **1** | This is a 65 year old male with type 2 diabetes and diabetic neuropathy. He was found to have a 1.7 g/dl monoclonal protein, IgA kappa, and a free light chain kappa of 178 mg/dl, and a ratio of 112. Hemoglobin is 8.5 g/dl and serum creatinine is 2 mg/dl. What is the diagnosis, and what should he be treated with? Pay attention to co-existing co-morbidities when making the treatment recommendation. |
| **2** | You are seeing a 56 year old female with relapsed multiple myeloma after 2 lines of therapy. She is triple class refractory. What are FDA approved treatment options for her? |
| **3** | You admitted a 68 year old male to the hospital with renal failure. His creatinine is 2.5 mg/dl. Other labs include hemoglobin 8.4 g/dl. Lumbar spine XR shows a 2 cm osteolytic lesion in the L4 vertebra. What tests should be done to establish the diagnosis? |
| **4** | A 59 year old female with relapsed multiple myeloma is treated in your clinic for disease progression. You are planning to start on treated with isatuximab, carfilzomib, and dexamethasone per the IKEMA trial. What is the recommended dosing for carfilzomib from this trial? |
| **5** | A 35 year old man with newly diagnosed high risk multiple myeloma is receiving treatment with Dara-RVD for his multiple myeloma. He has a history of Type 2 Diabetes, and recently his glycemic control has dramatically worsened. Which drug is responsible, and what should be done with his treatment to modify for this. |
| **6** | A 78 year old man with HTN and diabetes has relapsed multiple myeloma after treatment with daratumumab lenalidomide and dexamethasone. What are the approved options for treatment of his multiple myeloma in 2nd line? |
| **7** | A 56 year old man with triple class refractory multiple myeloma is relapsing 2.5 years after treatment with BCMA CAR T cells (cilta-cel). What are some FDA approved options for treatment that he could consider? |
| **8** | You are treating a 67 year old man with acute renal failure due to myeloma cast nephropathy. His creatinine is 2.5 mg/dl and his involved free light chain is 2500 mg/dl. You admit him to the hospital for hydration and treatment and plan to start a bortezomib based regimen. What is the best option for treatment and what dosing/frequency should be used? |
| **9** | A 59 year old female is seen in your clinic for relapsed multiple myeloma. She was diagnosed in 2017 and underwent treatment with RVD followed by autologous stem cell transplant in November 2017, then on lenalidomide maintenance. What are the FDA approved options for treatment? |
| **10** | A 72 year old man with CHF and chronic kidney disease has newly diagnosed multiple myeloma. He has an EF of 30% and creatinine of 3 mg/dl. Would he be a good candidate for autologous stem cell transplantation? |
| **11** | A 63 year old woman with multiple myeloma is seen in clinic while on treatment with Dara RVD. She has worsening pain and numbness in both legs bilaterally. Which of the drugs she is receiving is likely causing this, and what should be done? |
| **12** | A 54 year old man with relapsed multiple myeloma is treated with carfilzomib, daratumumab, and dexamethasone. He starts noticing lower extremity edema and worsening dyspnea. Which drug could be causing this, and what is the diagnostic work up? |
| **13** | A 71 year old female with relapsed multiple myeloma has had 4 lines of therapy and has received bortezomib, lenalidomide, daratumumab, and carfilzomib. Which bispecific antibodies that are FDA approved could she receive? |
| **14** | A 78 year old female with newly diagnosed multiple myeloma, with Deletion 17p, is seen in clinic to discuss treatment options. What are the options for treatment of non-transplant eligible multiple myeloma, newly diagnosed, that are FDA approved in the US? |
| **15** | A 63 year old female with relapsed multiple myeloma after autologous stem cell transplantation and lenalidomide maintenance is seen in clinic. Which CAR T cell therapy is approved after 1 line of therapy, and what is the median PFS for this product? |
| **16** | A 75 year old male with Parkinson's and Diabetes has newly diagnosed multiple myeloma. What are the best treatment options, which are FDA approved, and based on randomized phase 3 trials? |
| **17** | A 45 year old male with newly diagnosed multiple myeloma is revised ISS stage 3, and has t(14;16) and del17p by FISH. Following autologous stem cell transplant, what should he receive for maintenance given his high risk features? |
| **18** | A 64 year old male with newly diagnosed multiple myeloma is on hemodialysis for chronic kidney disease. You would like to start him on Dara RVD. What dose of lenalidomide should be used from the package insert? |
| **19** | A 39 year old male with relapsed multiple myeloma is awaiting CAR T cell therapy. He is triple class refractory. Leukapheresis is not for 8 weeks. His myeloma is progressing. What are treatment options that could be considered to hold his disease? |
| **20** | A 60 year old female with newly diagnosed multiple myeloma has 25% circulating plasma cells in the peripheral blood. What are treatment options for plasma cell leukemia based on randomized phase 3 trials? |
| **21** | A 73 year old male with newly diagnosed multiple myeloma is ineligible for autologous stem cell transplantation. What are FDA approved options for treatment of newly diagnosed MM? |
| **22** | A 32 year old male with standard risk multiple myeloma is seen after autologous stem cell transplant. Based on phase 3 trials, what are options for maintenance therapy? |
| **23** | A 52 year old female with high-risk multiple myeloma (t(4;14) and del17p) underwent Dara KRD induction and autologous stem cell transplant. Given her high risk disease, what should she receive for maintenance, and for how long? |
| **24** | A 78 year old male with HTN and Parkinson's has newly diagnosed multiple myeloma. What are approved options for treatment of non-transplant eligible MM in this patient? |
| **25** | A 66 year old female with newly diagnosed MM underwent treatment with Dara RVD and achieved a VGPR. Should she undergo a stem cell transplant? She is otherwise in good health. |
| **26** | A 68-year-old male with high-risk relapsed/refractory multiple myeloma (RRMM), complicated by hypertension, type 2 diabetes, and chronic kidney disease (eGFR 53 ml/min). His initial treatment included bortezomib, lenalidomide, and dexamethasone without transplant, resulting in a partial response lasting 8 months before he became refractory. The second line, with carfilzomib, pomalidomide, and dexamethasone, also achieved a partial response for 4 months, after which he became refractory again. The third line treatment, comprising daratumumab, carfilzomib, and lenalidomide, yielded no response, and the fourth line with selinexor, pomalidomide, and dexamethasone also resulted in no response. What is the recommended next line of treatment? |
| **27** | A 68-year-old male with high-risk relapsed/refractory multiple myeloma (RRMM), with additional comorbidities of hypertension and type 2 diabetes. His initial treatment was bortezomib, lenalidomide, and dexamethasone with a transplant, leading to a partial response for 8 months before becoming refractory. In the second line, he received daratumumab, carfilzomib, and dexamethasone, achieving a very good partial response lasting 6 months. The third line, involving daratumumab, carfilzomib, and lenalidomide, led to a partial response for 4 months before becoming refractory. His fourth line treatment with cilta-cel resulted in a complete response lasting 18 months. What is the recommended next line of treatment? |
| **28** | A 62-year-old female with standard-risk relapsed/refractory multiple myeloma (RRMM) and cardiovascular disease, including angina but no history of myocardial infarction. She initially received bortezomib, lenalidomide, and dexamethasone with a transplant, achieving a complete response that lasted 60 months. Upon becoming refractory, she was treated with carfilzomib, lenalidomide, and dexamethasone, resulting in a very good partial response for 6 months. What is the recommended next line of treatment? |
| **29** | A 70-year-old male with high-risk relapsed/refractory multiple myeloma (RRMM), complicated by diabetes and obesity and harboring the t(4;14) translocation. His initial treatment included bortezomib, lenalidomide, and dexamethasone with a transplant, leading to a very good partial response that lasted 10 months before he became refractory. The second line, involving daratumumab, bortezomib, and dexamethasone, resulted in a partial response lasting 5 months. The third line with elotuzumab, pomalidomide, and dexamethasone yielded a minimal response for 3 months. What is the recommended next line of treatment? |
| **30** | A 59-year-old female with standard-risk relapsed/refractory multiple myeloma (RRMM) and a history of asthma and osteoporosis. She received daratumumab, lenalidomide, bortezomib, and dexamethasone, achieving a complete response lasting 15 months. After becoming refractory, she was treated with carfilzomib, pomalidomide, and dexamethasone, resulting in a very good partial response for 7 months. The third line, including selinexor, bortezomib, and dexamethasone, yielded no response. The fourth line with cilta-cel achieved a complete response lasting 24 months. What is the recommended next line of treatment? |
| **31** | A 75-year-old male with high-risk relapsed/refractory multiple myeloma (RRMM) due to del(17p), complicated by chronic kidney disease (eGFR 53 ml/min) and hypertension. His first treatment regimen with daratumumab, lenalidomide, and dexamethasone without transplant resulted in a very good partial response lasting 9 months before he became refractory. The second line with carfilzomib, pomalidomide, and dexamethasone yielded a partial response for 5 months. The third line involving isatuximab, bortezomib, and dexamethasone resulted in no response, followed by a fourth line with talquetamab, which achieved a partial response lasting 8 months. What is the recommended next line of treatment? |
| **32** | A 64-year-old female with standard-risk relapsed/refractory multiple myeloma (RRMM) and comorbidities of hypertension and hyperlipidemia. Her initial treatment with bortezomib, lenalidomide, and dexamethasone, along with a transplant, led to a complete response lasting 50 months. In the second line, she received daratumumab, carfilzomib, and dexamethasone, achieving a complete response that lasted 25 months. The third line, including elotuzumab, pomalidomide, and dexamethasone, resulted in a minimal response for 3 months. The fourth line with Abecma yielded a very good partial response lasting 10 months. What is the recommended next line of treatment? |
| **33** | A 75-year-old male with high-risk relapsed/refractory multiple myeloma (RRMM) due to the t(14;16) translocation, and a history of cardiovascular disease with a myocardial infarction 2 years prior to his myeloma diagnosis, as well as type 2 diabetes. His initial treatment with daratumumab, lenalidomide, and dexamethasone without transplant achieved a complete response lasting 33 months before he became refractory. In the second line, he was treated with pomalidomide, bortezomib, and dexamethasone, leading to a very good partial response for 8 months. The third line treatment with selinexor, bortezomib, and dexamethasone resulted in no response. What is the recommended next line of treatment? |
| **34** | A 58-year-old female with standard-risk relapsed/refractory multiple myeloma (RRMM) and a history of osteoporosis and diabetes. She initially received daratumumab, lenalidomide, bortezomib, and dexamethasone with a transplant, achieving a complete response lasting 78 months. After becoming refractory, she was treated with carfilzomib, pomalidomide, and dexamethasone, resulting in a very good partial response for 26 months. The third line treatment with cilta-cel led to a complete response lasting 27 months. What is the recommended next line of treatment? |
| **35** | A 69-year-old male with high-risk relapsed/refractory multiple myeloma (RRMM) due to the t(4;14) translocation, and additional comorbidities including chronic kidney disease (eGFR 72 ml/min), obesity, and hypertension. His initial treatment with lenalidomide, bortezomib, and dexamethasone with a transplant resulted in a very good partial response lasting 58 months. The second line with daratumumab, pomalidomide, and dexamethasone yielded a partial response for 9 months. The third line, involving selinexor, bortezomib, and dexamethasone, led to stable disease. What is the recommended next line of treatment? |
| **36** | A 63-year-old female with high-risk relapsed/refractory multiple myeloma (RRMM) complicated by cardiovascular disease (no history of myocardial infarction) and hypertension, and harboring the del917p deletion. Her initial treatment with bortezomib, lenalidomide, and dexamethasone with a transplant led to a partial response lasting 23 months before she became refractory. The second line treatment with daratumumab, bortezomib, and dexamethasone resulted in a partial response lasting 7 months. The third line with elotuzumab, pomalidomide, and dexamethasone yielded a minimal response for 3 months, followed by a fourth line with selinexor, bortezomib, and daratumumab, which achieved no response. What is the recommended next line of treatment? |
| **37** | A 76-year-old male with high-risk relapsed/refractory multiple myeloma (RRMM) due to the t(14;16) translocation and additional comorbidities including diabetes and chronic kidney disease, with a history of cerebrovascular accident while on lenalidomide maintenance. His initial treatment with bortezomib, lenalidomide, and dexamethasone without transplant led to a partial response lasting 15 months before he became refractory. The second line treatment with pomalidomide and dexamethasone resulted in a minimal response for 2 months. What is the recommended next line of treatment? |
| **38** | A 65-year-old female with standard-risk relapsed/refractory multiple myeloma (RRMM) and comorbidities of osteoporosis, mild hypertension, and type 2 diabetes. Her first treatment regimen with daratumumab, bortezomib, lenalidomide, and dexamethasone with a transplant achieved a complete response lasting 63 months. What is the recommended next line of treatment? |
| **39** | A 48-year-old male with high-risk relapsed/refractory multiple myeloma (RRMM) due to del(17p). His initial treatment with daratumumab, bortezomib, lenalidomide, and dexamethasone with a transplant resulted in a partial response lasting 23 months before he became refractory. What is the recommended next line of treatment? |
| **40** | A 41-year-old male with relapsed/refractory multiple myeloma (RRMM) with high risk cytogenetic (del(17p)). No other health issues. He was initially treated with bortezomib, lenalidomide, and dexamethasone with transplant, achieving a complete response that lasted 45 months. Upon relapse, he was treated with daratumumab, pomalidomide, and dexamethasone, resulting in partial response for 3 months. Patient is interested in receiving CAR-T cells. Is CAR-T cell recommended for this patient at this time and why? If CAR-T cell is recommended what specific CAR-T is recommended and why? |
| **41** | A 73-year-old male with relapsed/refractory multiple myeloma (RRMM) with high risk cytogenetic (del(17p)). Past medical history includes (i) coronary artery disease with history of MI 3 years ago, currently LVEF of 52%, (ii) hypertension controlled with appropriate treatment, (iii) type 2 diabetes well controlled. For his MM he was initially treated with daratumumab, lenalidomide and dexamethasone, achieving very good partial response that lasted 37 months. Upon relapse, he was treated with carfilzomib, pomalidomide, and dexamethasone, resulting in partial response for 3 months. Patient is interested in receiving CAR-T cells. Is CAR-T cell recommended for this patient at this time and why? If CAR-T cell is recommended what specific CAR-T is recommended and why? |
| **42** | A 53-year-old female with triple class refractory multiple myeloma, previously treated with daratumumab-Revlimid-bortezomib-dexamethasone followed by autologous stem cell transplant, she relapsed after 62 months and was treated with carfilzomib-pomalidomide and dexamethasone, but with no response. She has no other medical problems. She is now a candidate for CAR-T cell therapy with cilta-cell. Should the patient receive bridging therapy between apheresis and CAR-T cell infusion and with what regimen? |
| **43** | A 67-year-old female with standard disk cytogenetics triple class refractory multiple myeloma after 4 prior lines of therapy. Past medical history significant for renal insufficiency with creatinine of 2.1 mg/dL, hypertension well controlled, and type 2 diabetes not well controlled with HgA1C of 6.7. For next line treatment for multiple myeloma would you recommend CAR-T cells or T-Cell-Engager (bispecific antibody) and why? Whatever class of treatment you recommend, may you please also recommend the specific treatment and the rationale for the recommendation? |
| **44** | What is the recommended treatment for the following patient with newly diagnosed multiple myeloma: A 65-year-old male with high-risk multiple myeloma (del(17p), t(14;16)), suffering from COPD and hypertension. |
| **45** | What is the recommended treatment for the following patient with newly diagnosed multiple myeloma: A 62-year-old female with standard-risk multiple myeloma, who has hyperlipidemia and Type 2 diabetes. |
| **46** | What is the recommended treatment for the following patient with newly diagnosed multiple myeloma: A 70-year-old male with high-risk multiple myeloma (t(4;14), del(17p)), suffering from chronic kidney disease (eGFR 52 ml/min) and heart failure (ejection fraction 40%). |
| **47** | What is the recommended treatment for the following patient with newly diagnosed multiple myeloma: A 58-year-old female with intermediate-risk multiple myeloma (t(11;14)), who has asthma and hypothyroidism. |
| **48** | What is the recommended treatment for the following patient with newly diagnosed multiple myeloma: A 75-year-old male with standard-risk multiple myeloma, also diagnosed with diabetes and peripheral vascular disease. |
| **49** | What is the recommended treatment for the following patient with newly diagnosed multiple myeloma: A 66-year-old female with high-risk multiple myeloma (del(17p)), who has a history of coronary artery disease with a myocardial infarction 2 years prior to the diagnosis of multiple myeloma, along with Type 2 Diabetes mellitus. |
| **50** | What is the recommended treatment for the following patient with newly diagnosed multiple myeloma: A 68-year-old male with high-risk multiple myeloma (del(17p)), also suffering from Type 2 Diabetes mellitus and hypertension. |

**Recency-sensitive annotation**

To address reviewer feedback, we added a post-hoc descriptive label indicating whether accurate answers for each scenario required clinical evidence that became available within 12 months before the June 1, 2024 cutoff. This annotation was not part of the original scenario design and does not influence any model outputs or rater evaluations.

Because updates in multiple myeloma therapeutics occur on a continuum and often lack a single definitive cutoff, the classification is based on best clinical judgment and should be interpreted as an approximate indicator rather than a definitive binary category. The recency-sensitive labels are provided in Supplementary Table S1 for transparency and interpretability and are not used for inferential analyses.

Table S1. Recency-Sensitive Scenarios

| **Scenario** | **1** | **2** | **3** | **4** | **5** | **6** | **7** | **8** | **9** | **10** |
| --- | --- | --- | --- | --- | --- | --- | --- | --- | --- | --- |
| Recency-Sensitive | F | T | F | F | F | T | T | F | T | F |
| **Scenario** | **11** | **12** | **13** | **14** | **15** | **16** | **17** | **18** | **19** | **20** |
| Recency-Sensitive | F | F | T | T | T | T | F | F | T | F |
| **Scenario** | **21** | **22** | **23** | **24** | **25** | **26** | **27** | **28** | **29** | **30** |
| Recency-Sensitive | T | F | F | T | F | T | T | T | T | T |
| **Scenario** | **31** | **32** | **33** | **34** | **35** | **36** | **37** | **38** | **39** | **40** |
| Recency-Sensitive | T | T | T | T | T | T | T | T | T | T |
| **Scenario** | **41** | **42** | **43** | **44** | **45** | **46** | **47** | **48** | **49** | **50** |
| Recency-Sensitive | T | T | T | T | T | T | F | T | T | T |

**1. T = TRUE (recency-sensitive)**

**2. F = FALSE (not recency-sensitive)**

# **C. Additional Results**

We report domain-stratified performance for five clinical domains: Diagnostic, NDMM, RRMM, Special clinical scenarios, and Novel therapeutics/special populations. For each model within each domain, we summarize the mean scores for accuracy, relevance, and comprehensiveness (4-point scales averaged across raters and scenarios), the rates of hallucination and ready-to-use (binary outcomes reported as proportions), and the sample sizes: $N_{question}$(distinct scenarios in the domain) and $N_{ratings}$=$N_{question}\times3$ (three raters per scenario). Aggregation follows the main Methods: three hematologist-oncologists independently scored each response; domain-level means and rates were then computed across all responses produced by a model within that domain. Because some domains contain a modest number of scenarios, we report $N_{question}$​ and $N_{ratings}$ alongside every estimate to aid interpretation.

Table S2. Domain-level performance of five LLMs across multiple myeloma scenarios

| Group | LLM | N_evals | Accuracy | Relevance | Comprehensiveness | Hallucination | Ready_to_use |
| --- | --- | --- | --- | --- | --- | --- | --- |
| Diagnostic | ChatGPT o1-preview | 6 | 2.67 | 2.83 | 2.67 | 0.00 | 0.00 |
| Diagnostic | Claude 3.5 Sonnet | 6 | 3.00 | 2.67 | 2.83 | 0.00 | 0.17 |
| Diagnostic | Gemini 1.5 Pro | 6 | 2.17 | 2.33 | 2.50 | 0.17 | 0.17 |
| Diagnostic | HopeAI | 6 | 3.17 | 3.17 | 2.83 | 0.00 | 0.33 |
| Diagnostic | Myelo | 6 | 2.83 | 3.17 | 2.83 | 0.00 | 0.17 |
| NDMM | ChatGPT o1-preview | 45 | 2.78 | 2.71 | 2.22 | 0.02 | 0.13 |
| NDMM | Claude 3.5 Sonnet | 45 | 2.62 | 2.62 | 2.29 | 0.11 | 0.07 |
| NDMM | Gemini 1.5 Pro | 45 | 2.53 | 2.40 | 2.00 | 0.04 | 0.07 |
| NDMM | HopeAI | 45 | 3.22 | 3.27 | 3.13 | 0.04 | 0.38 |
| NDMM | Myelo | 45 | 2.56 | 2.60 | 2.16 | 0.02 | 0.02 |
| Novel/SpecialPop | ChatGPT o1-preview | 15 | 2.87 | 2.67 | 2.53 | 0.07 | 0.07 |
| Novel/SpecialPop | Claude 3.5 Sonnet | 15 | 2.87 | 2.53 | 2.27 | 0.00 | 0.13 |
| Novel/SpecialPop | Gemini 1.5 Pro | 15 | 2.60 | 2.53 | 2.27 | 0.20 | 0.00 |
| Novel/SpecialPop | HopeAI | 15 | 3.07 | 3.00 | 2.87 | 0.07 | 0.40 |
| Novel/SpecialPop | Myelo | 15 | 2.87 | 2.73 | 2.40 | 0.00 | 0.07 |
| RRMM | ChatGPT o1-preview | 63 | 2.76 | 2.65 | 2.25 | 0.05 | 0.11 |
| RRMM | Claude 3.5 Sonnet | 63 | 2.40 | 2.48 | 2.16 | 0.11 | 0.02 |
| RRMM | Gemini 1.5 Pro | 63 | 2.33 | 2.25 | 1.94 | 0.10 | 0.03 |
| RRMM | HopeAI | 63 | 3.08 | 3.22 | 3.10 | 0.06 | 0.32 |
| RRMM | Myelo | 63 | 2.49 | 2.44 | 2.10 | 0.06 | 0.02 |
| Special | ChatGPT o1-preview | 21 | 2.81 | 2.67 | 2.52 | 0.00 | 0.19 |
| Special | Claude 3.5 Sonnet | 21 | 2.62 | 2.71 | 2.52 | 0.14 | 0.14 |
| Special | Gemini 1.5 Pro | 21 | 2.86 | 2.71 | 2.43 | 0.00 | 0.19 |
| Special | HopeAI | 21 | 3.05 | 2.95 | 2.90 | 0.05 | 0.24 |
| Special | Myelo | 21 | 3.05 | 2.95 | 2.67 | 0.14 | 0.29 |

Besides, we conducted a domain-stratified comparison (accuracy, rank-based). Within NDMM (15 scenarios), a Friedman test across five systems indicated significant differences (χ²(4)=18.605, p=0.00094). Average ranks (lower=better) were: HopeAI 1.67, o1-preview 2.77, Claude 3.43, Gemini 3.43, Myelo 3.70; with Nemenyi’s critical difference CD=1.576, HopeAI ranked significantly ahead of Claude, Gemini, and Myelo, whereas the margin vs o1-preview did not exceed the CD. Within RRMM (21 scenarios), differences were also significant (χ²(4)=41.192, p=2.45×10⁻⁸). Average ranks were HopeAI 1.43, o1-preview 2.64, Myelo 3.26, Claude 3.57, Gemini 4.10; with CD=1.331, HopeAI outperformed Myelo, Claude, and Gemini, and o1-preview outperformed Gemini; HopeAI vs o1-preview was not beyond the CD. For classes with fewer scenarios (Special 7; Novel/SpecialPop 5; Diagnostic 2), we report descriptive summaries in the table above only, given limited power for formal inference.

**References**

[1] Ferber, D., Wiest, I. C., Wölflein, G., Ebert, M. P., Beutel, G., Eckardt, J. N., ... & Kather, J. N. (2024). GPT-4 for Information Retrieval and Comparison of Medical Oncology Guidelines. NEJM AI, 1(6), AIcs2300235.

[2] Chen, S., Kann, B. H., Foote, M. B., Aerts, H. J., Savova, G. K., Mak, R. H., & Bitterman, D. S. (2023). Use of artificial intelligence chatbots for cancer treatment information. JAMA oncology, 9(10), 1459-1462.

[3] Low, Y. S., Jackson, M. L., Hyde, R. J., Brown, R. E., Sanghavi, N. M., Baldwin, J. D., ... & Gombar, S. (2024). Answering real-world clinical questions using large language model based systems. arXiv preprint arXiv:2407.00541.

[4] International Myeloma Foundation Launches Myelo: The First AI-Powered Responsive Chatbot to Provide Compassionate Myeloma Support for Patients, Care Partners, and Healthcare Professionals. myeloma.org
